# Linguistic processes do not beat visuo-motor constraints, but they modulate where the eyes move regardless of word boundaries: Evidence against top-down word-based eye-movement control during reading

**DOI:** 10.1371/journal.pone.0219666

**Published:** 2019-07-22

**Authors:** Claire Albrengues, Frédéric Lavigne, Carlos Aguilar, Eric Castet, Françoise Vitu

**Affiliations:** 1 Université Côte d’Azur, CNRS, BCL (Bases, Corpus, Langage), Nice, France; 2 Lab by MANTU, Amaris Research Unit, Biot, France; 3 Aix-Marseille Univ, CNRS, LPC (Laboratoire de Psychologie Cognitive), Fédération de Recherche 3C, Marseille, France; Nagoya University, JAPAN

## Abstract

Where readers move their eyes, while proceeding forward along lines of text, has long been assumed to be determined in a top-down word-based manner. According to this classical view, readers of alphabetic languages would invariably program their saccades towards the center of peripheral target words, as selected based on the (expected) needs of ongoing (word-identification) processing, and the variability in within-word landing positions would exclusively result from systematic and random errors. Here we put this predominant hypothesis to a strong test by estimating the respective influences of language-related variables (word frequency and word predictability) and lower-level visuo-motor factors (word length and saccadic launch-site distance to the beginning of words) on both word-skipping likelihood and within-word landing positions. Our eye-movement data were collected while forty participants read 316 pairs of sentences, that differed only by one word, the prime; this was either semantically related or unrelated to a following test word of variable frequency and length. We found that low-level visuo-motor variables largely predominated in determining which word would be fixated next, and where in a word the eye would land. In comparison, language-related variables only had tiny influences. Yet, linguistic variables affected both the likelihood of word skipping and within-word initial landing positions, all depending on the words’ length and how far on average the eye landed from the word boundaries, but pending the word could benefit from peripheral preview. These findings provide a strong case against the predominant word-based account of eye-movement guidance during reading, by showing that saccades are primarily driven by low-level visuo-motor processes, regardless of word boundaries, while being overall subject to subtle, one-off, language-based modulations. Our results also suggest that overall distributions of saccades’ landing positions, instead of truncated within-word landing-site distributions, should be used for a better understanding of eye-movement guidance during reading.

## Introduction

Reading is a complex perceptual and cognitive task, that not only involves the identification of individual words and their integration in the sentences' syntactic and semantic context, but also requires the execution of saccadic eye movements along the lines of text. Necessitated by the strong decrease of visual acuity with retinal eccentricity, saccades play a crucial role in that they determine which letters and words benefit from detailed viewing on successive eye fixations. Yet, whether they are in turn cognitively guided towards the center of target words (or target word-objects), as selected based on the (expected) needs of ongoing word-identification processing, still remains an open question. This is a long-standing assumption, that accounts for a number of well-established eye-movement phenomena (e.g. [1–4]). Nevertheless, given the slowness of language-related processes and top-down guidance, the possibility remains that saccades primarily reflect low-level visual and oculomotor processes, that make no recourse to selection of a saccade-target word(-object) [5–8]. Here we further challenged the top-down word-based view by re-examining the respective influences of visual and linguistic variables on where the eyes move during reading, and testing in particular one of its strong predictions: that linguistic factors should exclusively influence the likelihood a word is fixated (vs. skipped), and not where in a word the eyes land, rather than overall modulating saccade amplitudes regardless of word boundaries.

The hypothesis that eye movements during reading are guided in a top-down, word-based, manner was originally proposed towards the mid-seventies (e.g. [9]), and it has since then been a predominant assumption, being expressed in different variants, ranging from strategy-based guidance to language-based guidance (e.g. [10–13]). It remains today a central assumption, that is implemented in the great majority of models of eye-movement control during the reading of alphabetic languages ([1–4], see also [14–16], but see [17–18]), and to some extent also during Chinese reading ([19–20], but see [21]). Although word-based models differ in several important ways, most rely on the same three basic principles, as originally proposed by McConkie and colleagues [22]: (1) On every eye fixation, a word(-object) is designated as the next-saccade target; (2) The functional target location is the center of the word, to optimize subsequent visual-information uptake and word identification ([23]; for a review see [24]), although this may shift towards the beginning of words, when the level of uncertainty associated with the currently fixated word (N) is high, as proposed in SERIF [3], or when word segmentation cannot be achieved, as may occur during the reading of unspaced Chinese text materials [20]; (3) Where the eyes effectively land results from a compromise between this (word-center) targeting strategy and both systematic saccadic range error (SRE [25–26], but see [27–28]), a bias to move the eyes a constant, optimal, distance forward (see also [29]), and random error.

Word-based models also share the assumption that selection of the saccade target word depends on the (estimated) efficiency of letter-extraction and/or word-identification processes, weighted by visual acuity. Where these models differ is mainly in the processing stages that enable this selection. In E-Z Reader, words are identified sequentially based on successive attention shifts [2]. The target word is by default the next word (N+1) on the line, and a saccade to that word starts being programmed as soon as the fixated word (N) has reached a preliminary stage of word processing (i.e., word-familiarity check). However, when Word N is identified, attention shifts towards Word N+1, enabling in turn its processing in peripheral vision; if the word-familiarity check associated with this word is complete before the saccade program enters a non-labile stage, Word N+2 becomes the saccade target, and Word N+1 is skipped. In SWIFT and GLENMORE, words are processed in parallel within the perceptual span [1, 4]. The target corresponds to the word whose processing-based "saliency" is the highest by the time a random saccade timer, or the level of fixation activity, possibly combined with language-related inhibition, enables the programming of a saccade. The selected word thus depends on the amount of lexical processing achieved on foveal and peripheral words by the time a saccade is ready to go. However, for early-triggered saccades, as additionally proposed in GLENMORE, it is purely determined based on letter visibility; the word-object with the highest letter-based saliency becomes the target of the next saccade. Finally, in SERIF, the saccade target is a blob; it is determined in a probabilistic manner, based on the chances of identifying the words within the right/forward perceptual span, as inferred from the words’ length and eccentricity, as well as their frequency in the language [3].

Regardless of the processing stages involved, these models all make the same general predictions. As they all rely on the general hypothesis that saccades invariably aim for the center of selected target words (but see [3, 20]), with systematic and random errors being the only source of variability, they predict that a word's linguistic properties should nearly exclusively influence the likelihood the word is skipped, but not where in the word the eyes initially land. Due to SRE, within-word landing-position distributions should progressively shift towards the very-end of words as saccades are launched from closer to the words’ beginning, in line with the well-established launch-site effect ([22, 30]; for Chinese reading see [20, 31]). However, they should not be affected by the easiness of peripheral word processing, except maybe as a result of word-skipping failure, thus in the very rare instances when a word, intended to be skipped, would end up being fixated due to systematic and/or random errors. Still, as mislocated fixations would mainly lay towards the very-end of words [32, 33], only the tail of landing-site distributions could possibly diverge between easy and difficult words.

The central hypothesis in word-based models, that readers’ eye movements reflect word(-object)-based saccade-targeting mechanisms combined with SRE, however remains debatable. First, as suggested by alternative, visual-(perceptual-)span models, a continuous (non-word-based), rather than a discrete (word-based), adjustment of saccades to the needs of ongoing visual and lexical word-identification processes could also yield seemingly word-based eye-movement behavior (e.g., the skipping of shorter and easier words), as well as a launch-site effect ([17, 34, 35], see also [36]; for Chinese reading see [21]). More critically, as suggested by several empirical findings, and in contradiction with word-based, as well as non-word-based visual-/perceptual-span, models, both language-related processes and top-down selection of a saccade goal may be too much time consuming to be the main eye-driving force [5–8]. Moreover, a low-level visuo-motor account for the launch-site effect may be more appropriate than either SRE or processing-based explanations ([28, 37–39], see also [27]). Vitu’s [5–6, 40] bottom-up, non-word-based, Center-of-Gravity (CoG) theory of eye-movement guidance during reading relies on these two assumptions, and as we will see, this yields radically different predictions in comparison with word-based models. According to this view, where the eyes move when proceeding forward along the lines of text, would primarily reflect low-level spatial-integration mechanisms involved in saccade programming (for reviews see [5, 41]). By averaging over spatially proximal, bottom-up, luminance-contrast signals, within and across word boundaries, these mechanisms would take the eyes towards a fovea-weighted center of gravity of the peripheral configuration formed by letters ahead of fixation, regardless of their identity and the word they belong to. Thus, as saccades are launched from closer to the words’ beginning, and even more so as the words are shorter, the eyes would land further on the line of text, and hence closer to the words’ end or even beyond it, neither as a result of SRE nor ongoing processing, but simply because of non-word-based spatial-integration processes. In this framework, ongoing visual and lexical peripheral word-identification processes would also intervene. However, given the poor resolution in peripheral vision combined with the slowness of language-related processes [42, 43], they would only mildly modulate default saccade amplitude, and only in particular instances, i.e., when the words’ visual and linguistic properties combine to favor an early access to the word’s representation, and/or when fixations are prolonged.

**Fig 1:**
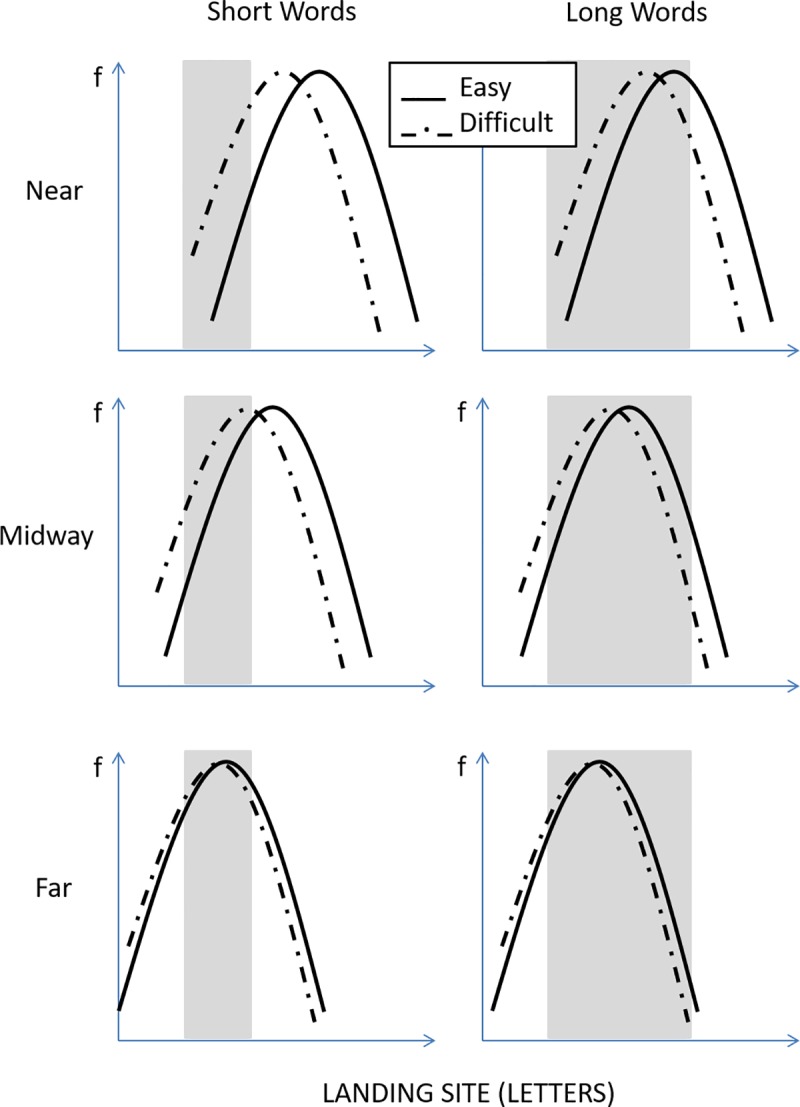
Illustration of the predictions made by the bottom-up, non-word-based, CoG hypothesis. Under this assumption, saccade amplitudes should be overall modulated by word-processing difficulties regardless of word boundaries, but to greater extents for shorter and less eccentric words, that more greatly benefit from peripheral preview. Are represented the hypothetical frequency distributions of saccades’ landing positions on the line of text for easy (plain lines) vs. difficult (dotted lines) peripheral words (N+1), separately for short and long words (left and right panels), and close, intermediate, and far saccadic launch-site distances to the beginning of the words (from upper to lower panels). Light-grey rectangle areas represent the horizontal extent of the words. Landing positions falling within those areas correspond to within-word landing positions, while landing positions to the right of these areas result in word skipping.

The bottom-up, non-word-based, CoG theory, thus predicts that a word's linguistic properties could potentially, though only mildly, influence not only the likelihood the word is skipped, but also where in the word the eyes initially land. However, while in theory language-related variations in word-skipping rate and within-word landing positions should both become greater as the words are shorter and less eccentric (and more letters from the words fall within the perceptual span), they should in fact be observed for words of different lengths and/or for different saccadic launch-site distances respectively. This is illustrated in Fig 1, where we represented a hypothetical overall slight shift of landing-site distributions towards the end of easy words, in comparison with difficult words, for different word lengths and saccadic launch-site distances to the beginning of words; the implemented shift was slightly greater for shorter (left panels) and less eccentric words (upper panels) to reflect the fact that these words more greatly benefit from peripheral preview. This figure suggests that significant effects of word difficulty could potentially be observed on within-word landing positions, but less likely on word-skipping rate, when the distributions happen to peak near the center of words, thus when the launch-site distance is sufficiently large and/or words are long enough for the processing-related shift in landing-site distributions to take place within the word boundaries (see left lower panel and all three right panels). Since these are not all optimal conditions for peripheral word processing, these effects would yet remain rather small, and potentially difficult to observe. In contrast, when the distributions peak near the end of words or even beyond it, as in the case of shorter and less eccentric words (which are also more easily processed), the shift would most often occur outside the word boundaries, and likely result in a significant effect of word difficulty on the likelihood of word skipping, but not on within-word landing positions (see left upper and middle panels). Thus, in this specific case, the non-word-based hypothesis would meet the predictions of word-based models, but for different reasons. Note though that language-related effects should remain much smaller than the effects of word length and saccadic launch-site distance, that would essentially result from earlier spatial-integration mechanisms [5, 6]. Top-down, word-based (and non-word-based), models, and E-Z Reader and SWIFT [1–2] in particular, which (unlike GLENMORE [4]) do not assume different time courses for visual and lexical processes respectively, may yield a different prediction, at least with no proof to the contrary.

At present, there is no unambiguous evidence for either word-based or non-word-based predictions. In line with both views, previous studies on the reading of alphabetic, as well as un-spaced non-alphabetic, languages revealed that words are more likely to be skipped when they are shorter (e.g. [9, 20]), and/or nearer to the saccade’s starting location (or launch site [44, 45]), as well as when they benefit from peripheral preview [46–49], they are more frequent in the language ([20, 50–56], but see [48, 57]), and/or they can be more easily predicted from the sentence's context ([20, 48, 50, 55, 58–65]; for reviews see [11, 44, 66–68]). Moreover, as predicted by word-based and non-word-based accounts, word skipping rate was found to be more greatly affected by word frequency and word predictability, when saccades were launched from closer to the words' beginning [69–70], or the words could benefit from peripheral preview [21]. It still remains uncertain how top-down models, and E-Z Reader and SWIFT in particular, would cope with the likely greater variations in word-skipping rate with word length, compared to word frequency or word predictability, as reported in two meta-analyses ([44, 66], see also [54, 71], but see [72]), and as further suggested by comparison of normal reading and the reading of meaningless, z-transformed, texts ([73–75], see also [76]).

More critically, although many studies on the reading of alphabetic languages failed to show variations in within-word landing positions with peripheral-preview manipulations [28, 77, 78], or the frequency and the predictability of words [56, 61, 62, 69, 70, 79–84], some studies did reveal small though significant effects of word frequency [55, 56, 85], and/or word predictability [86, 87]. Moreover, a great deal of experiments showed tiny, though consistent, effects of orthographic ([82, 83, 88–94], but see [52, 70, 95]) and morphological word properties ([56, 85, 96–103], but see [83, 91, 104–105]) on within-word landing positions. Importantly, these effects, as well as word-frequency effects, were reported mainly in long words (> = 7 letters on average), and they held across the entire range of landing positions at least in studies reporting landing-position distributions [56, 82, 83, 88, 91, 93, 101], thus in line with predictions from the non-word-based hypothesis. In a similar manner, Lavigne, Vitu and d'Ydewalle [86] observed an overall slight shift of within-word landing-position distributions towards the end of predictable words, in comparison with non-predictable words, that held only for high-frequency target words of 6–8 letters, in intermediate launch-site conditions (> = -7 letters from the words' beginning). Still, Rayner and colleagues [69] found only a hint of an effect of word predictability towards the very-end of 5- and 6-letter words in close launch-site conditions (> = -4 letters from the beginning of words), but a significant effect on the likelihood of word skipping. This could well be evidence for (assuming their words were too short) or against non-word-based guidance.

Likewise, several studies on the reading of Chinese text material revealed small though significant effects of peripheral preview and/or word frequency on within-word landing positions [21, 31, 48–49, 106], while others showed non-significant effects for words of comparable length (2 characters) [48, 107]. Importantly though, Liu and colleagues [21] observed that peripheral-preview and word-frequency effects not only held over the entire range of within-word landing positions [108], but also generalized to saccades’ landing positions on the line, thus within and across words’ boundaries (what we refer to as overall landing positions [39]), as well as forward saccade amplitude. While their findings more convincingly argue for non-word-based guidance, the question remains whether this would be specific to the reading of un-spaced non-alphabetic languages.

The problem with most previous studies is that they were not optimally designed to provide a strong test of the above, word-based and non-word-based, predictions. The number of items per frequency and/or predictability classes was often relatively low, and hence made it difficult to further split the data by word length (when this was manipulated) and launch site. Moreover, the discretization of the independent variables, for the needs of the analyses (ANOVAS in the great majority of studies; but see [21, 53, 54, 56, 63, 85]), was probably not optimal to capture likely subtle and complex trends. The present study overcame these limitations by re-investigating the relative influence of word frequency, word predictability, word length and saccadic launch-site distance on both within-word initial landing sites and word-skipping rate, using (generalized) linear-mixed-effect modeling applied to a large corpus of eye-movement data. This corpus, referred from now on to as the “French-sentence corpus”, was collected while 40 adult participants each read a total of 316 sentences. As in Lavigne et al.'s [86] original study, word predictability was manipulated by using pairs of sentences, that were strictly identical, except for the prime word that was either semantically related or unrelated to a subsequent test word, making a total of 632 sentences. The semantic relatedness between prime and test words was estimated based on the association strength between the two words, as measured in free production norms; the predictability of the test words in the sentences was further assessed using a cloze task. Across sentences, the test word was of variable frequency and length.

Both word-based and non-word-based hypotheses predicted that the likelihood of skipping the test words would vary with their frequency and predictability, though more greatly for shorter and less eccentric words, and hence when visual, lexical and semantic peripheral-word information would get together for a faster access to the word's representation. However, while word-based models predicted that within-word initial landing positions should not be significantly affected by the words’ frequency and predictability, the non-word-based, view predicted frequency and predictability effects, but mainly for longer words, and/or intermediate launch-site distances (see Fig 1). Moreover, only the bottom-up non-word-based assumption did unambiguously predict that language-related variations in both word-skipping rate and within-word landing positions would remain much smaller than the effects of word length and saccadic launch-site distance.

## Materials and methods

### Participants

Forty ***s***tudents (between 20 and 30 years old) from Aix-Marseille University were paid 15€ to participate in the experiment. All were native speakers of French and had normal and uncorrected vision. None was aware of the goal of the experiment. Participants gave their written informed consent prior to their participation in the experiment, that was conducted in accordance with the ethical standards laid down in the Declaration of Helsinki. This research was approved by the committee responsible for overseeing research conducted in human subjects at Aix-Marseille University (Comité d’éthique de l’université d’Aix-Marseille; Pierre-Jean Weiller, President).

### Materials

A total of 316 pairs of sentences, containing 31–69 characters (mean: 50.40, SD: 7.31) and 6–14 words (mean: 9.21, SD: 1.43), were constructed. Each contained both a prime and a test word, with the prime word appearing first, at the second position in the sentences, and the test word appearing on average 2.8 words later, though never being last, or preceded or followed by punctuation. The two sentences of a given pair were matched except for the prime word which was either semantically related or unrelated to the test word. In each pair, related and unrelated primes were matched in length up to a two-letter difference.

Related prime and test words were selected from available free word-production norms in French [109, 110]; for these, participants were asked to produce the first (test) word (e.g., ‘volcano’) that came to their mind when reading a given (prime) word (e.g., ‘lava’). The computed association strength between the two words corresponded to the proportion of participants producing the test word given the prime. For the 316 related word pairs that were selected for the sentences, the test word was related to the prime with a strength greater than 0.01 (*M* = 0.36, *SD* = 0.20; range from 0.01 to 0.91). For the 316 corresponding control sentences, using the same test words but a different prime, the association strength between prime and test words was 0.

To control for the predictability of the test words in the sentences’ context, and hence not only relative to the prime, a preliminary study was conducted using a cloze task. In this study, a total of 92 participants (all French-native speakers) were asked to indicate which word first came to their mind when reading the beginning of each of the 632 sentences (up to the word before the test word). This allowed us to calculate the proportion of participants producing the test word in each sentence. In sentences containing related word pairs, and hence predictable sentences, the test word was given by 22–100% of the participants (*M* = 0.66, SD = 0.23), while it was given by 0–4% of the participants (*M* = 0,005, *SD* = 0.013) in corresponding unrelated-word-pair (or unpredictable) sentences (see examples *a* and *b*).

*a*. La *lave* s’échappe du *volcan* en éruption (predictability = 0.83)
    *Lava* is escaping from a *volcano* in eruption
*b*. La *fumée* s’échappe du *volcan* en éruption (predictability = 0.00)
    *Smoke* is escaping from a *volcano* in eruption

All selected test words were between 2 and 13 letters long (*M* = 6.05 letters, *SD* = 1.97 letters), and had a frequency between 0 and 1,289 occurrences per million (*M* = 59.31, *SD* = 129.88, according to the variable “Freqlvr” in lexique.org [111]). More details on the distribution of word lengths, word frequencies, and word predictabilities across test words is given in Table 1. In comparison, the range of word frequencies across all words in the sentences was much larger (0.07–38930 occurrences per million; *M*: 6128.00; *SD*: 9676.45); these words were 1–13 letters long (*M*: 4.58; *SD*: 2.61).

**Table 1:** Properties of the test words.

		WORD FREQUENCY				WORD PREDICTABILITY							
						Non-Predictable Sentences				Predictable Sentences			
WORD													
LENGTH	N	Min	Max	M	SD	Min	Max	M	SD	Min	Max	M	SD
2	1	127.23	127.23	127.23	/	0.00	0.00	0.00	/	1.00	1.00	1.00	/
3	20	1.76	315.74	91.58	93.93	0.00	0.04	0.01	0.01	0.26	1.00	0.66	0.24
4	53	0.00	861.49	88.52	164.82	0.00	0.04	0.01	0.01	0.22	1.00	0.62	0.23
5	69	0.14	1289.39	85.07	209.45	0.00	0.04	0.01	0.01	0.22	1.00	0.72	0.23
6	52	0.20	328.78	47.83	63.29	0.00	0.04	0.00	0.01	0.22	1.00	0.68	0.24
7	48	1.22	343.72	52.98	74.82	0.00	0.04	0.00	0.01	0.22	1.00	0.63	0.23
8	41	0.54	73.38	17.18	21.94	0.00	0.04	0.00	0.01	0.22	0.91	0.64	0.20
9	18	0.34	73.38	20.57	22.72	0.00	0.04	0.00	0.01	0.22	1.00	0.69	0.23
10	6	0.74	37.36	13.75	15.52	0.00	0.00	0.00	0.00	0.27	0.96	0.63	0.29
11	5	0.54	15.95	5.11	6.21	0.00	0.04	0.01	0.02	0.30	0.96	0.63	0.26
13	3	0.68	5.68	3.20	2.50	0.00	0.00	0.00	0.00	0.43	0.57	0.51	0.07

From left to right, for each test word length: the number of words, the minimum (Min), maximum (Max), mean (M) and standard deviation (SD) of the words’ frequency (in occurrences per million), and the minimum (Min), maximum (Max), mean (M) and standard deviation (SD) of the words’ predictability (expressed as a proportion) in non-predictable and predictable sentences respectively.

For the Latin-square design (see below), the 632 sentences were divided into two sub-lists, each containing a total of 316 sentences; half of these sentences were predictable, and the other half were unpredictable, but only one exemplar (predictable or unpredictable) of a sentence pair was present in a given sub-list.

### Design

Length, frequency and predictability of the test word were manipulated, using a repeated-measure design. Saccades' launch-site distance to the space in front of the test words was defined a posteriori. In the analyses, all four variables were defined as continuous predictors (see Data selection and analyses). Each participant saw only one of the two sub-lists of 316 sentences (see Materials), meaning that he/she saw all test words, but only once, either in the predictable or in the unpredictable condition. However, all 632 sentences were seen across all participants (Latin-square design). For the experiment, each of the two sub-lists was split into six blocks balanced in predictability, frequency and length. The first two blocks contained 60 sentences. The third, fourth, fifth and sixth blocks contained 54, 50, 49 and 43 sentences, respectively. In each block, the order of the sentences was randomized.

### Procedure

Upon arrival, the participant was seated comfortably in front of a computer screen, with his/her head movements being minimized with a bite-bar and a frontal head rest. Then, a 15-point calibration phase took place, with the dot appearing successively at 15 positions on the screen (along the two diagonals and above and below the horizontal midline, where the sentence would be further displayed). The participant was asked to first fixate the dot in the upper left corner of the screen, as accurately as possible. When he/she estimated that his/her eyes correctly fixated the dot, he/she pressed a button, which made the point disappear and reappear at the next screen location. The calibration phase was repeated until the correlation between the position of the dot and the estimated eye location was greater than 0.99. A block of trials was then launched.

At the beginning of each trial in a block, the participant was asked to fixate in between two vertically aligned bars presented in the left part of the screen, and centered on the horizontal midline, where a sentence would next be displayed. When a fixation was detected within a circular region of 0.5° radius around the bars, the sentence appeared. This remained on screen until the participant indicated through key press that he/she was done with the reading of the sentence. In 20% of the cases, that were distributed randomly within a block, a yes/no comprehension question was then displayed; this was related to the sentence the participant had just read. Participants pressed the right button for a "yes" response, and the left button for a “no” response. After a delay of 2000 ms, the next trial began.

Participants were given a block of 30 practice trials followed by a total of six blocks of test trials. Participants were allowed to take a pause whenever they wanted in between the blocks. Each session lasted approximately 1 hour and 30 min.

### Apparatus

Eye movements were recorded using a 5th generation Dual-Purkinje-Image (DPI) Eye-Tracker (Ward Technical Consulting), sampling the right eye position every millisecond with a spatial accuracy of 10 min of arc [112]. The eye tracker was connected through a National-Instruments (USB 6221 multifunction card) converter to an Intel Xeon dual-core computer running Windows XP. The computer was connected to two screens (one for the experimenter and one for the participant). Custom software was developed with the NI LabVIEW ® 2009 Integrated Development Environment to acquire and analyze the eye-movement signal online; this software also controlled the presentation of the stimuli, contingent on the position of the eye. The eye-position signal was re-analyzed offline, using the offline saccade/fixation detection algorithm developed by Engbert and Kliegl [113] and implemented in the R software [114] by Laubrock and Kliegl (eyetrackR package; in prep.). Sentences were displayed in white on a black background. They were written in lower cases, except for the first letter of the first word in the sentences as well as the first letter of proper nouns, using the fixed-width *Courier-New* font in PsychoPy. Sentences were saved as separate bitmaps, that were displayed on a gamma-corrected 21” CRT monitor with 85-Hz refresh rate and a screen resolution set to 1280 x 960 pixels. At a distance of 118 cm from the participants' eyes, each character subtended about 0.25 degrees of visual angle. The room was dark except for a dim indirect light source. Vision was binocular.

### Data selection and analyses

In the first, main, set of analyses, we measured the likelihood of skipping the test word, as well as the initial eye fixation location in the test word, when this was fixated. We then extended these analyses to all words in the sentences that responded to a number of selection criteria. In both sets of analyses, the fixation of interest was the very first fixation on the space, or beyond the space, in front of a given word (the test word in the main set of analyses). This fixation was selected when (1) it was not preceded or followed by a blink or any signal irregularity, (2) it was within 1° above or below the screen midline where the sentence was displayed, and it was preceded by a fixation also within these vertical margins, (3) it was not the last fixation on the line, and the immediately prior fixation was not the first fixation on the line, (4) it was preceded by a forward saccade, and (5) it corresponded to the very-first fixation on a word. In analyses related to the test word, it was further ensured that the prime word had received at least one fixation before fixation on the test word or past it (i.e., when the test word was skipped during the first eye pass). In analyses that were not restricted to the test word, additional selections were applied to keep only the words that were neither the first nor the last in a sentence, and that were not preceded or followed by punctuation; compound words were also filtered out.

Within-word landing positions were analyzed by fitting linear mixed-effect models (LMM) to the data, using the *lmer* function from the *lme4* package (Version 1.1–7 [115]) in R (Version R-3.1.3 [114]). Binary, word-skipping, data were fitted with Generalized LMM (GLMM), using the *glmer* function. The models were implemented after visualizing the data and checking for the linearity of the relationships between the dependent variables and each of the predictors, as well as between the predictors. When linearity was not justified due to a few extreme predictor values being associated with a low *n* (e.g., log word frequency < = 0 in word-skipping analyses), these were filtered out to avoid making the model too complex by adding polynomial components, and running the risk in turn that the model would not converge or would give unrealistic estimates. Furthermore, to avoid modeling floor/ceiling effects, further selections were applied to the data. In word-skipping analyses, the words that were either very short or very long and too far out in the periphery were filtered out, as these were associated respectively with one- and zero-skipping probabilities in many participants. In within-word landing position analyses, extreme launch-site values were removed because these were associated mostly with landing positions outside the word boundaries, and hence within-word landing positions that no longer varied with launch-site distance.

To determine the (G)LMM that best fitted our data, a top-down approach was used, that consisted of first determining the optimal random structure, using the most complex fixed structure, and then searching for the optimal fixed structure, given the optimal random structure [116]. The starting fixed structure included a linear component for each predictor (word length, launch-site distance, word frequency, and also word predictability in test-word analyses), and all interactions, though never four-way interactions; the latter are indeed difficult to interpret and actually often prevented GLMM convergence. The optimal random structure was determined after comparing the goodness of fit of a range of models varying in random structures, using Akaike’s information criterion (AIC); the model with the smallest AIC was selected. The range of tested random structures comprised a random intercept by participant and/or sentence pair (and/or word number in list, in analyses that were not restricted to the test word), with or without by-participant random effects of each (possible combination) of the predictors, and with or without the correlation between random effects; random effects by sentence pair and/or word number were not included for simplicity (for a similar approach and further justifications see [117]). The optimal fixed structure was determined after dropping successively the predictors, from the higher to the lower-order terms (3-way interactions first, and then 2-way interactions, and then simple effects), that did not significantly improve the fit of the model; note that when the removal of a given predictor only marginally significantly improved the fit, the predictor was kept. Importantly, when a given interaction needed to be kept, corresponding lower-order terms (interactions and simple effects) were also kept regardless of whether or not removing them would improve the fit of the model (for a similar approach see [56]). This made fixed-effects tables easier to read, and to compare with theoretical predictions: as simple effects provide an estimate of the dependent variable when all predictors are at their reference value, they contribute to describe the observed interaction(s). However, since removing vs. keeping lower-order terms is a matter of debate, minimalist optimal (G)LMM were also determined by applying the dropping procedure to all predictors, regardless of whether, or not, higher-order terms were kept. When the optimal fixed structure of minimalist optimal (G)LMM differed from the fixed structure of optimal (G)LMM, the fixed effects of the former were reported in Supporting Information. Note though that the fixed-effects’ estimates were quite comparable between minimalist optimal models and optimal models (see Tables 2–8 for comparison); the only notable difference was for the models presented in Table 3 and S3 Table, as mentioned in the main text. For both optimal and minimalist-optimal models, fixed and random structures were described in the tables’ captions. To represent graphically the estimated fixed effects from optimal (G)LMM, partial effects were computed, using the *ggpredict* function from the *ggeffects* (Version 0.8.0) package in R (Version R-3.5.3).

All predictors were defined as continuous variables; they were centered on their mean. Word frequency was expressed in log units, as classically done (e.g. [118]). For word predictability, expressed as a proportion, we used, following Kliegl et al. [118], the logit transform; logits were defined as 0.5*ln(predictability/(1-predictability)), but after replacing predictabilities of zero and 1 with 1/(2*92) and (2*92–1)/(2*92) respectively, where 92 represents the number of participants in the cloze task (see Materials). For saccadic launch-site distance, it is classically expressed in letters relative to the center of words, at least in analyses of within-word landing positions [22]. However, since our analyses were aimed at testing the general prediction that frequency and predictability combine with letter visibility in determining where the eye moves, defining launch-site distance relative to the space in front of the words was more appropriate. Indeed, for a given launch-site distance relative to the beginning of a word, but not relative to the center of the word, the number of letters falling within the perceptual span is the same irrespective of the word’s length. For illustration purposes only (but not for LMM analyses), word frequency (in log units), word predictability (in logit units), and launch-site distance (in letters) were categorized into two, three or four bins depending on the needs of the analyses; this was done after splitting the distribution of the corresponding variable in 2–4 equal parts respectively. Note that for word frequency, binning was made separately for different word lengths, given the correlation between word frequency and word length (target words: -0.20565; all words in the sentence: -0.56557, respectively).

The exact number of degrees of freedom for the t-values of fixed effects in LMMs remains undetermined. However, given the large number of observations, participants, and items entering our analyses, t-distributions converged to a normal distribution. Therefore, we considered as significant, the effects whose absolute t-value was greater than 2, which corresponds to a significance level of 5% in two-tailed tests [119, 120].

## Results

For comparison with previous reading studies, we first analyzed the global characteristics of our participants' eye movements while they were reading the sentences. As typically reported, we found that participants moved their eyes mainly forward, making regressions in about 14.94% of the cases on average [67]. The median length of their saccades was on average of about 8.35 and -4.49 letters, depending on whether they took their eyes forward or backward, while the median duration of their fixations was on average of about 241 ms. Participants skipped about 52.15% of the words on average during a first eye pass, and they refixated words (or made more than one consecutive fixation on a word) in about 11.38% of the cases on average.

We next tested alternative predictions from word-based and non-word-based accounts of eye guidance during reading. To this end, we analyzed the metrical properties of forward eye-movement behavior in the vicinity of the words (either the test words only or all words in the sentences that responded to our selection criteria–see Materials and Methods), using the likelihood of word skipping and (within-word) initial landing positions as dependent variables. These were analyzed as a function of saccadic launch-site distance to the space in front of the words, word length and word frequency, as well as word predictability in analyses restricted to the test words.

### Probability of skipping the test words

**Fig 2:**
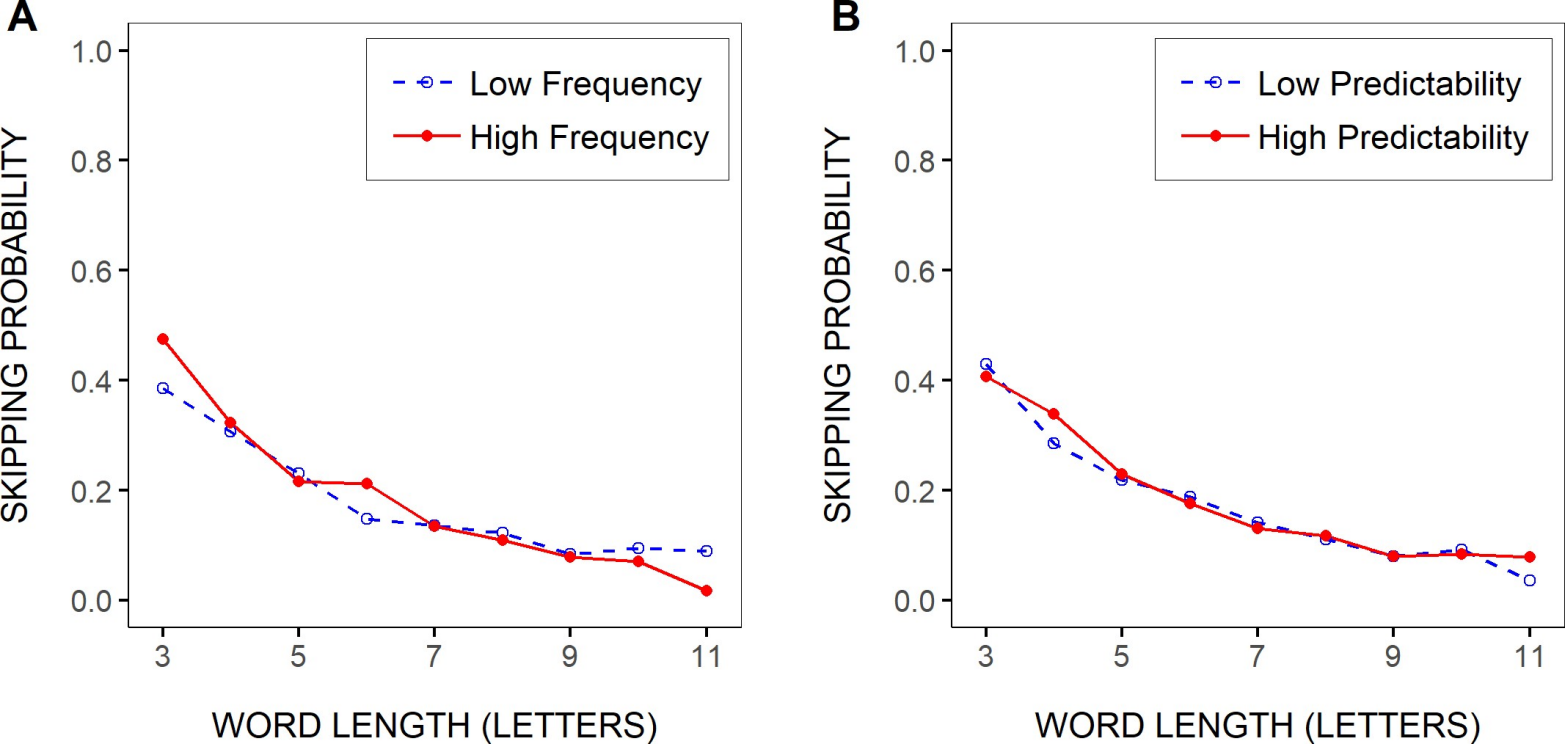
Test-word skipping rate by length, frequency and predictability. Mean probability of skipping the test words as a function of the words’ length (in letters), separately for two categories of test-word frequencies (A) and predictabilities (B), as determined after grouping test-word frequencies and predictabilities into two bins respectively (see Materials and Methods).

In Fig 2, the mean probability of skipping the test words was represented as a function of the words’ length, separately for two categories of word frequency and word predictability. This indicates that the likelihood of word skipping largely decreased with increasing word length but showed very little variation with language-related variables, being only slightly lower for low- compared to high-frequency words of 3–4 and 6 letters, and for low- compared to high-predictability words of 4 letters.

**Fig 3:**
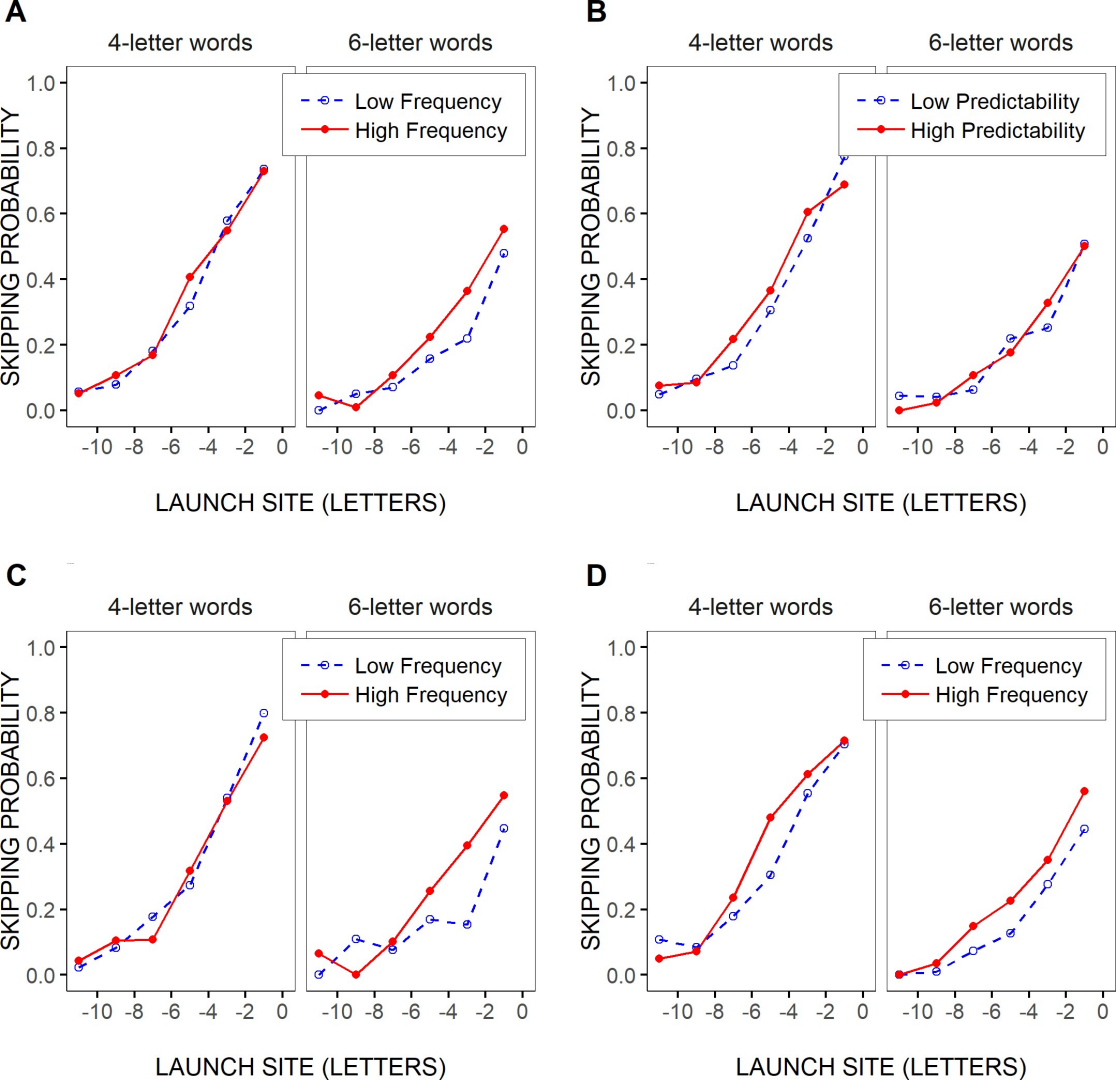
Test-word skipping rate by launch site, frequency and predictability. Mean probability of skipping 4- and 6-letter test words as a function of the saccades’ launch-site distance to the space in front of the words (binned in two-letter intervals), separately for two categories of test-word frequencies (across word predictabilities; A) and two categories of test-word predictabilities (across word frequencies; B), and for high- vs. low-frequency test words of low- and high-predictability (C and D respectively). The two categories of word frequencies and word predictabilities were determined after grouping test-word frequencies and predictabilities into two bins respectively (see Materials and Methods).

When data were further split by saccades’ launch-site distance to the space in front of the test words, the effects of linguistic variables tended to be clearer and more consistent, despite the lower *n*. This is shown in Fig 3A and 3B for the case of 4- and 6-letter words. Word-skipping rate was slightly lower for rare compared to more frequent words, as well as for low- compared to high-predictability words of 4 letters at least, though mainly in close launch-site conditions (> -8 letters). Moreover, there was a trend for the effect of word frequency to be slightly greater in high- compared to low-predictability words (Fig 3C and 3D). Yet, word-skipping rate remained more largely affected by word length and saccadic launch-site distance: as saccades were launched from further away from the beginning of the test words, the likelihood of word skipping decreased drastically, and even more so as words became longer.

Due to floor and ceiling effects, the respective and combined influences of the four independent variables on word-skipping likelihood could only be estimated over a subset of word lengths and saccadic launch-site distances. Therefore, to estimate the relationship between word skipping rate and word length, and its possible variations with word frequency and word predictability, nearly over the entire range of word lengths, a first GLMM (Model 1) was implemented, with only word length (3–11 letters), word frequency, word predictability, and their interactions, as predictors, thus across all observed saccadic launch-site distances. A second GLMM (Model 2), that included word length, saccadic launch-site distance, word frequency and word predictability, as well as all 3-way interactions, as predictors, was then fitted to a smaller subset of the data (word lengths between 4 and 8 letters and saccadic launch-site distances less than or equal to 6 letters from the space in front of the test words).

The fixed effects of Model 1 are presented in Table 2. The intercept estimate (logit: -1.59408), indicates that the test words were skipped in about 17% of the cases when all variables were at their reference (mean) value, and hence when the words were about 6 letters long. Shorter, 3-letter, test words were skipped about twice as often (37%), and longer, 11-letter, test words were skipped much more rarely (3%), as suggested by the significant negative slope estimate for the effect of word length (logit: -0.36063). There was no main effect of word frequency (logit: -0.01720, *p* = 0.50) or word predictability (this predictor and corresponding interactions were dropped from the fixed structure as they did not significantly improve the fit of the model). However, the significant negative slope estimate for the interaction between word frequency and word length (logit: -0.03300) suggested an increase in the effect of word length with increasing word frequency, implying that shorter words (i.e., less than about 6 letters, the reference, mean, value for word length) were skipped more often, and longer words were skipped less often, as they became more frequent.

**Table 2:** Fixed effects of optimal GLMM (Model 1) for the probability of skipping the test words.

	Estimate	Std. Error	z value	Pr(>|z|)
**(Intercept)**	-1.59408	0.08931	-17.84903	< 0.00001
**FREQ**	-0.01720	0.02532	-0.67921	0.49701
**LENGTH**	-0.36063	0.02753	-13.10137	< 0.00001
**FREQ:LENGTH**	-0.03300	0.01342	-2.45885	0.01394

The fixed structure included the effects of word length (“LENGTH”; 3–11 letters) and word frequency (“FREQ”; 0.20–5.93 log units), as well as their interaction; the random structure included a random intercept by participant and sentence pair, as well as a random effect of word length by participant (see S1 Table). The model's estimates and standard errors are expressed in logit units; they can be back transformed into probabilities, using the inverse logit formula. The intercept estimate (logit: -1.59408) indicates that the probability of word skipping was of about 0.17 when all variables were at their reference, mean, value (Word Length: 5.96 letters; Word Frequency: 3.03 log units; Predictability: -0.98 logit units). Colon stands for interaction. See S2 Table for the corresponding minimalist optimal GLMM.

**Fig 4:**
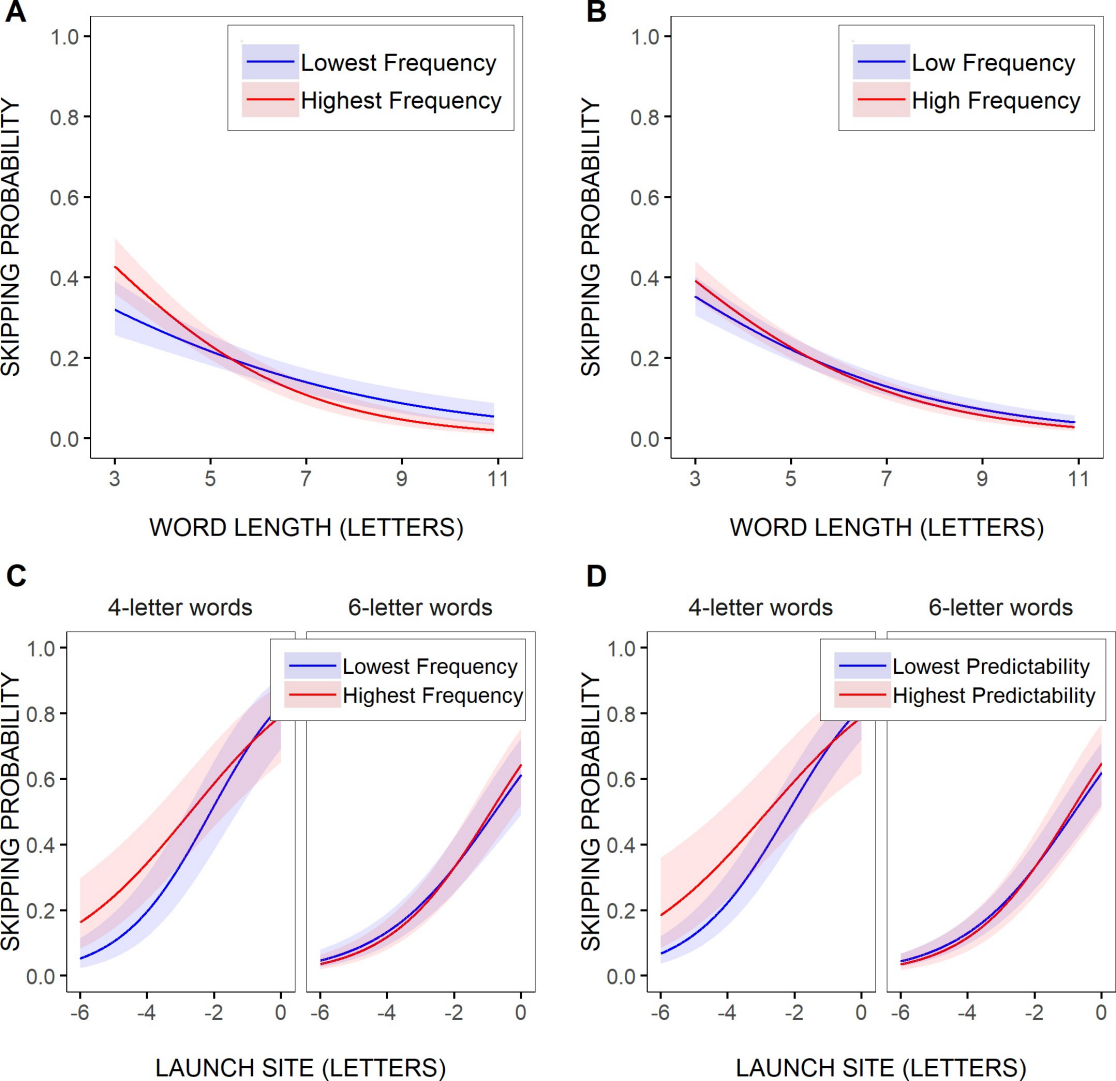
Estimated effects of visuo-motor and linguistic variables on test-word skipping rate. Partial effects (with 95% confidence intervals) computed from the parameters of GLMM Model 1 (A-B; Table 2) and GLMM Model 2 (C-D; Table 3), representing the estimated probability of skipping the test words as a function of word length (in letters; A-B), and for 4- and 6-letter test words as a function of saccadic launch-site distance (in letters relative to the space in front of the test words; C-D). In A,C, the models’ predictions were contrasted for the two most extreme (i.e., the lowest vs. the highest) word-frequency values across all selected test words regardless of their length and their predictability (0.20 and 5.93 log units respectively), and in D, they were contrasted for the two most extreme (i.e., the lowest vs. the highest) word-predictability values across all test words (-2.6 vs. 2.6 logit units). In B, Model 1’s predictions were represented for the mean frequency value of high vs. low-frequency words, as defined after grouping word frequencies into two bins (see Materials and Methods; 2.01 vs. 4.10 log units).

As further illustrated in Fig 4A, where the model’s predictions were represented for the two most extreme word-frequency values across all selected test words (0.20 vs. 5.93 log units), these variations in skipping rate with word frequency still remained very small in comparison with the effect of word length. The difference in word-skipping rate between the lowest and the highest word frequencies was of a maximum of about 11% in the shortest, 3-letter, test words, and this was yet an overestimation of the actual effect of word frequency, given the smaller range of word frequencies for most word lengths, as well as the variability in word frequencies. Indeed, when the model’s estimated word-skipping rate was contrasted for high- vs. low-frequency words on average (or the mean frequency of the test words, when categorized in two frequency bins), as in Fig 4B, the predicted effect was even tinier (see also Fig 2A). None of the other effects or interactions were significant.

As shown in Table 3, where Model 2’s fixed effects were reported, similar though clearer trends were observed when saccades’ launch-site distance relative to the space in front of the test words was taken into account. There was again a significant negative slope estimate for the effect of word length (logit: -0.46003), indicating that word-skipping rate decreased with increasing word length. In addition, there was a significant positive slope estimate for the effect of launch-site distance (logit: 0.61519), indicating that the test words were less frequently skipped as saccades were launched from further away from the words' beginning. Both effects were huge as word-skipping rate dropped by as much as 42% for a 6-letter increase in word length, and 60% for a 6-letter decrease in launch-site distance. Importantly, while there were again no significant effects of word frequency (logit: -0.00302, *p* = 0.94) and word predictability (logit: -0.00839, *p* = 0.73), the interaction between word frequency, word length, and launch-site distance was significant (logit: 0.02751), while the interaction between word predictability, word length and launch-site distance was marginally significant (logit: 0.01968, *p* = 0.09); note that the latter interaction was no longer significant in the minimalist optimal GLMM, that is when lower-order terms that did not significantly improve the fit of the model were removed (see S3 Table). As illustrated in Fig 4C and 4D, the estimated likelihood of skipping short, 4-letter, words slightly varied between the two most extreme word-frequency values across all test words, and to a lesser extent between the two most extreme word-predictability values, though essentially for large saccadic launch-site distances. In contrast, the difference in skipping rate between the highest and the lowest frequencies/predictabilities for longer, 6-letter, words was smaller, and it decreased with increasing launch-site distance. Still, even in 4-letter test words, the estimated frequency and predictability effects remained much smaller compared to the effect of launch-site distance (12% and 5% respectively compared to 72%).

**Table 3:** Fixed effects of optimal GLMM (Model 2) for the probability of skipping the test words.

	Estimate	Std. Error	z value	Pr(>|z|)
**(Intercept)**	-1.19934	0.14841	-8.08123	< 0.00001
**FREQ**	-0.00302	0.04398	-0.06872	0.94521
**PRED**	-0.00839	0.02461	-0.34099	0.73311
**LENGTH**	-0.46003	0.05747	-8.00517	< 0.00001
**LAUNCH**	0.61519	0.04106	14.98372	< 0.00001
**FREQ:LENGTH**	-0.04980	0.03213	-1.54996	0.12115
**FREQ:LAUNCH**	0.00729	0.01811	0.40250	0.68732
**PRED:LENGTH**	-0.02576	0.01987	-1.29634	0.19486
**PRED:LAUNCH**	0.01514	0.01428	1.06002	0.28914
**LENGTH:LAUNCH**	-0.00224	0.01905	-0.11767	0.90633
**PRED:LENGTH:LAUNCH**	0.01968	0.01150	1.71096	0.08709
**FREQ:LENGTH:LAUNCH**	0.02751	0.01339	2.05526	0.03985

The fixed structure included the effects of word length (“LENGTH”; 4–8 letters), word frequency (“FREQ”; 0.20–5.93 log units), word predictability (“PRED”; between -2.60 and 2.60 logit units), and saccadic launch-site distance (“LAUNCH”; between -6.00 and -0.002 letters from the space in front of the test words), the three-way interactions between word frequency, word length and launch-site distance and between word predictability, word length and launch-site distance, as well as corresponding two-way interactions; the random structure included a random intercept by participant and by sentence pair, as well as by-participant random effects of word length and launch-site distance, but without the correlation between random effects (see S1 Table). The model's estimates and standard errors are expressed in logit units. The intercept estimate (logit: -1.19934) indicates that test words were skipped in about 23% of the cases, when all variables were at their reference, mean, value (Word Length: 5.82 letters; Launch Site: -2.93 letters; Word Frequency: 3.06 log units; Word Predictability: -0.96 logit units). Colon stands for interaction. See S3 Table for the corresponding minimalist optimal GLMM.

In sum, the likelihood of skipping the test words was influenced by the words’ length and eccentricity, as well as the words’ linguistic properties. Yet, the effects of word length and saccadic launch-site distance predominated. They were not only greater in size compared to the effects of word frequency and word predictability, but they held nearly over the entire range of word frequencies and predictabilities. In contrast, word-frequency and word-predictability effects intervened only when the words were very short, and/or very close to the saccades’ launch site, thus when conditions were met for the words to benefit from peripheral preview. In other words, language-based word-skipping behavior seemed to emerge only when there was strong-enough evidence for the identity of the test word. The possibility remains that the small contribution of language-related variables was due to the specific (linguistic) properties of our test words, and their restricted range of frequencies. To ensure this was not the case, the same analyses were conducted again, but using this time all words in the sentences that could be possibly analyzed given our selection criteria.

### Skipping rate across all words in the sentences

The above analyses were restricted to the test words for the simple reason that test words were best controlled and differed not only in terms of their frequency in the language, but also their predictability from the sentence's context. However, the properties of the test words, and/or their relatively low n (see Table 1), could be responsible for our observation of a rather limited influence of language-related variables on word-skipping rate. Here, we thus replicated the above test-word skipping analyses, but using all words in the sentences, except for the words that did not respond to the above-defined selection criteria (see Materials and Methods). Note though that word predictability was not available for words other than the test words; it was therefore not considered in the present analyses.

**Fig 5:**
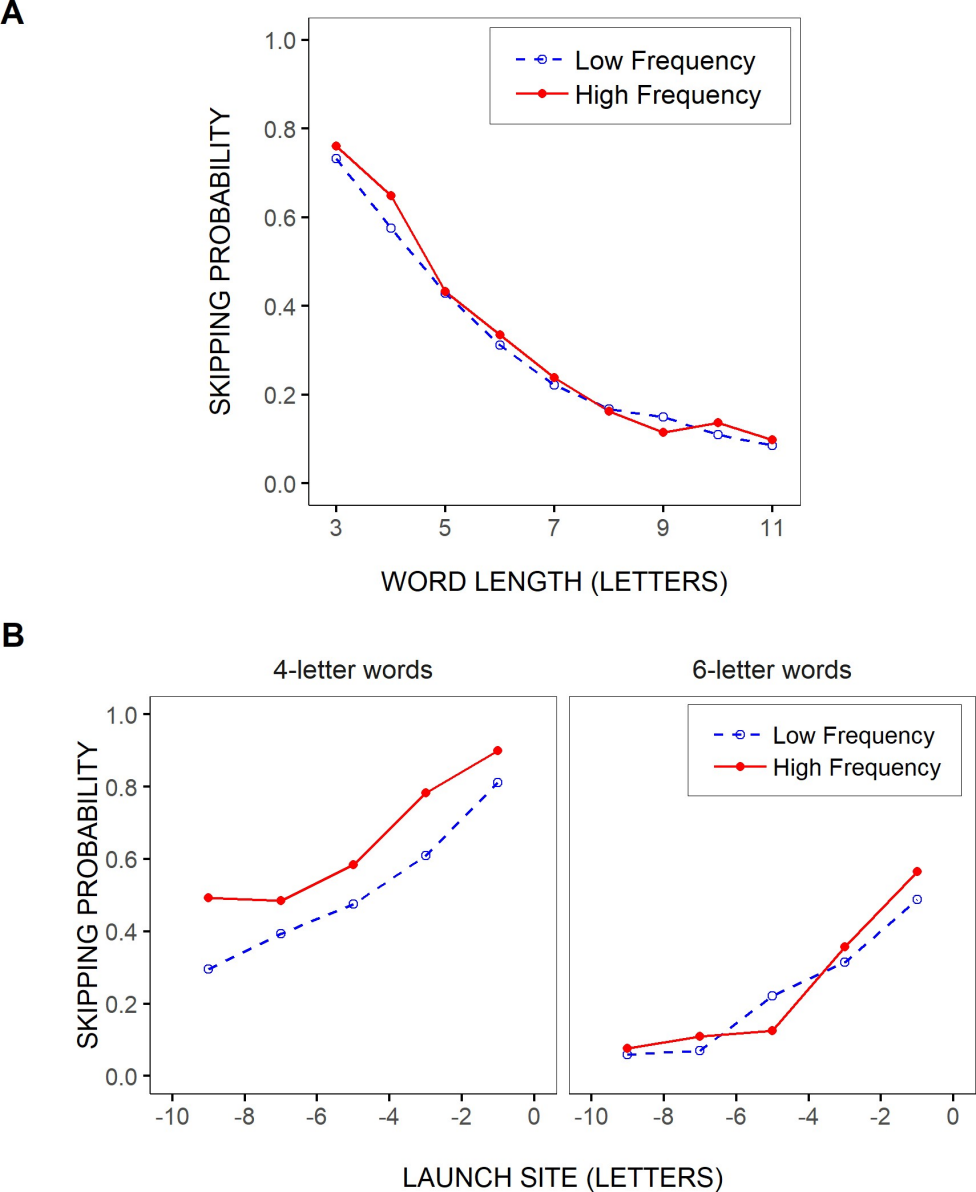
Word-skipping rate by length, launch site and frequency. Mean probability of word skipping, across all words in the sentences that responded to our selection criteria, as a function of word length (in letters; A), and for 4- and 6-letter words as a function of saccadic launch-site distance (in letters relative to the space in front of the words; B), separately for two categories of word frequencies, as determined after grouping word frequencies into two bins (see Materials and Methods).

As shown in Fig 5, word length and saccadic launch-site distance again predominated in determining the likelihood of word skipping. First, there was a gradual decrease in word-skipping rate with increasing word length, that largely remained unaffected by word frequency; only tiny differences between high- and low-frequency words emerged, and mainly for short, 3- and 4-letter, words (Fig 5A). Moreover, when data were further split by saccadic launch-site distance, separately for different word lengths, an effect of word frequency emerged, in addition to the drastic reduction in word-skipping rate with increasing launch-site distance, but mainly in short words (e.g., 4 letters; see Fig 5B). In longer, 6-letter, words, the effect was already strongly reduced, being visible only in very-near launch-site cases.

**Fig 6:**
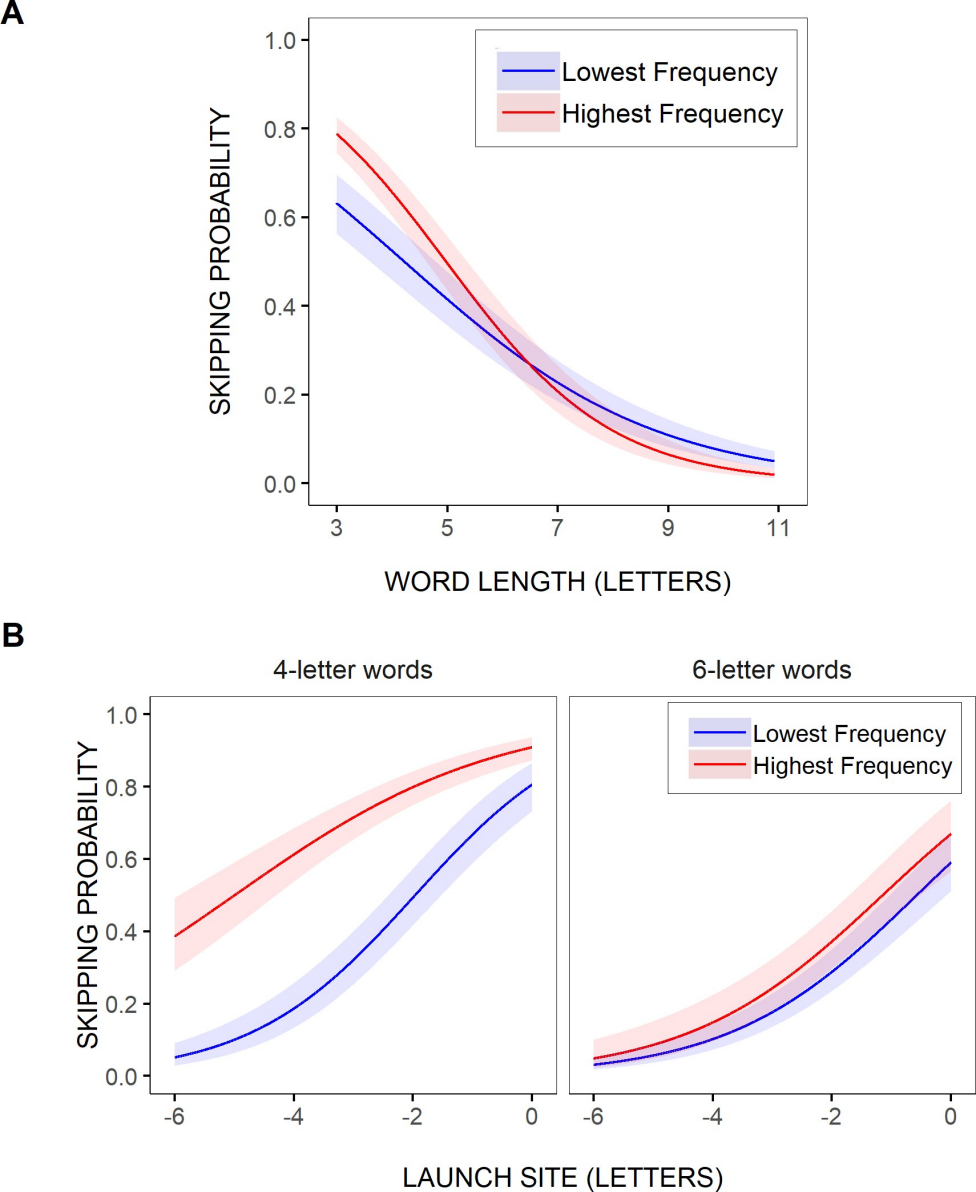
Estimated effect of visuo-motor and linguistic variables on word-skipping rate. Partial effects (with 95% confidence intervals) computed from the parameters of GLMM Model 1’ (A; Table 4) and GLMM Model 2’ (B; Table 5), representing the probability of word skipping across all words in the sentences as a function of word length (A) and for 4- and 6-letter words as a function of saccadic launch-site distance (in letters relative to the space in front of the words; B), separately for the two most extreme (i.e., the lowest vs. the highest) word-frequency values across all words selected for analysis (0.01 and 9.59 log units respectively).

To further test these trends, two GLMMs were fitted to the data, as for the test words. The first, Model 1’, tested the contribution of word length and word frequency, as well as their interaction, nearly over the entire range of word lengths (3–11 letters). As shown in Table 4, where the model’s fixed effects are reported, the likelihood of word skipping significantly decreased with increasing word length (logit: -0.56967). It also varied with word frequency (logit: 0.03371), being greater for higher-frequency words, though gradually less as the words were longer, as suggested by the significant interaction between word frequency and word length (logit: -0.02326). In fact, as illustrated in Fig 6A, where the model’s predicted relationship between word-skipping rate and word length was represented separately for the two most extreme word-frequency values across all selected words, the word-frequency effect held only for very short words. Moreover, as in the above test-word analyses, this effect was much smaller compared to the effect of word length: Word-skipping rate dropped by about 70% for an 8-letter reduction in word length (3–11 letters), while it varied by about 16% at the very most (i.e., for 3-letter words) between the highest and the lowest word frequencies.

**Table 4:** Fixed effects of optimal GLMM (Model 1’) for the probability of word skipping.

	Estimate	Std. Error	z value	Pr(>|z|)
**(Intercept)**	-0.16568	0.10549	-1.57054	0.11629
**FREQ**	0.03371	0.01432	2.35335	0.01861
**LENGTH**	-0.56967	0.02626	-21.69550	< 0.00001
**FREQ:LENGTH**	-0.02326	0.00473	-4.92211	< 0.00001

This analysis was conducted across all words in the sentences that responded to our selection criteria (see Materials and Methods). The fixed structure included the effects of word length (“LENGTH”; 3–11 letters) and word frequency (“FREQ”; between 0.01 and 9.59 log units), as well as the interaction; the random structure included a random intercept by participant, sentence pair, and word, as well as by-participant random effects of word length and word frequency, but without their correlation (see S1 Table). The model's estimates and standard errors are expressed in logit units. The intercept estimate (logit: -0.16568) indicates that the words were skipped in about 46% of the cases when all variables were at their reference, mean, value (Word Length: 5.02 letters; Word Frequency: 5.56 log units). Colon stands for interaction. The corresponding minimalist optimal GLMM was identical.

Model 2’ included saccadic launch-site distance, and its interaction with word length and/or word frequency, as additional predictors, but for a subset of the data given floor and ceiling effects (word lengths between 4 and 8 letters and launch-site distances less than or equal to 6 letters). As summarized in Table 5, there were again significant effects of word length (logit: -0.69301) and launch-site distance (logit:0.61995), indicating that the likelihood of word skipping strongly decreased as words became longer and saccades were launched from further away. In addition, there was a main effect of word frequency (logit: 0.06766), such that more frequent words were skipped more often. Still, this effect was again greater for shorter words, as suggested by the significant interaction between frequency and length (logit: -0.06234). Moreover, the three-way interaction between word frequency, word length and launch-site distance was significant (logit: 0.01349). This is illustrated in Fig 6B, where the model’s predicted relationship between word-skipping probability and saccadic launch-site distance was represented for the two most extreme word-frequency values across all selected words, separately for 4- and 6-letter words. As for the test words, there was an effect of word frequency in short 4-letter words, that held over the entire range of tested saccadic launch-site distances (> = -6 letters), but barely no frequency effect in longer, 6-letter, words, except maybe for very-small launch-site distances. This effect, even in 4-letter words where it was the largest, again remained much smaller than the effect of launch-site distance: for a 6-letter increase in launch-site distance, word-skipping rate decreased by about 71%, while it varied by a maximum of about 34% between the highest and the lowest word frequencies. Recall though that this was still an overestimation: given the variability in word frequencies, the actual effect of word frequency was even smaller (see Fig 5B).

**Table 5:** Fixed effects of optimal GLMM (Model 2’) for the probability of word skipping.

	Estimate	Std. Error	z value	Pr(>|z|)
**(Intercept)**	-0.69956	0.13511	-5.17778	< 0.00001
**FREQ**	0.06766	0.01676	4.03729	0.00005
**LENGTH**	-0.69301	0.04295	-16.13351	< 0.00001
**LAUNCH**	0.61995	0.04408	14.06404	< 0.00001
**FREQ:LENGTH**	-0.06234	0.01041	-5.98644	< 0.00001
**FREQ:LAUNCH**	-0.00768	0.01026	-0.74814	0.45438
**LENGTH:LAUNCH**	0.01414	0.01788	0.79096	0.42897
**FREQ:LENGTH:LAUNCH**	0.01349	0.00608	2.21757	0.02658

This analysis was conducted across all words in the sentences that responded to our selection criteria (see Materials and Methods). The fixed structure included the effects of word length (“LENGTH”; 4–8 letters), word frequency (“FREQ”; between 0.01 and 9.02 log units), and saccadic launch-site distance (“LAUNCH”; between -6.00 and -0.002 letters from the space in front of the test words), as well as all interactions; the random structure included a random intercept by participant, sentence pair, and word, as well as by-participant random effects of word length and saccadic launch-site distance (see S1 Table). The model's estimates and standard errors are expressed in logit units. The intercept estimate (logit: -0.69956) indicates that the words were skipped in about 33% of the cases, when all variables were at their reference, mean, value (Word Length: 5.60 letters; Launch Site: -2.40 letters; Word Frequency: 4.33 log units). Colon stands for interaction. See S4 Table for the corresponding minimalist optimal GLMM.

Thus, when all words in the sentences were considered for analysis, the pattern of findings matched that observed in test words. The likelihood of word skipping was again primarily influenced by word length and saccadic launch-site distance. Word frequency also contributed, but to a much smaller extent compared to visuo-motor variables, and mostly when the words could benefit from peripheral view, that is when they were very short or very-near to the saccade’s launch-site. These findings, consistent with both word-based and non-word-based accounts of eye-movement guidance, may still represent a challenge for models like E-Z Reader [2] and SWIFT [1] (see Discussion).

### Initial landing positions in the test words

Showing that word-skipping behavior is primarily a function of visuo-motor variables may represent a challenge for word-based models, and E-Z Reader [2] and SWIFT [1] in particular, as this clearly shows that eye-movement guidance from one word to the next cannot exclusively rely on ongoing word-identification processes. However, it does not necessarily challenge the hypothesis that saccades are guided in a top-down manner towards the center of selected target word-objects. Analyses of within-word landing positions were aimed at directly testing this assumption. These investigated whether the same variables that were found to influence word-skipping rate would also influence where in a word the eye lands, as would be predicted exclusively by a non-word-based account of eye-movement guidance.

**Fig 7:**
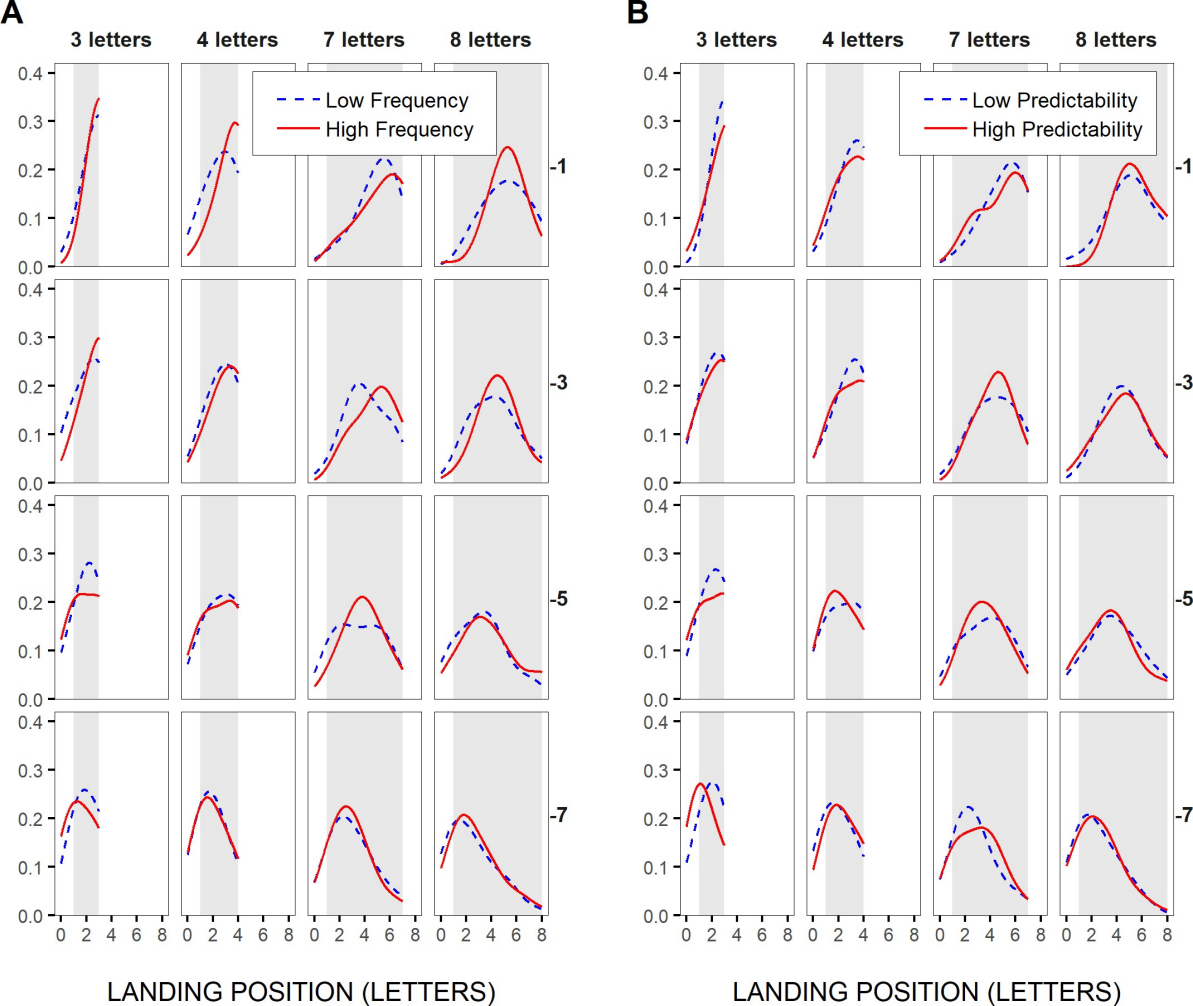
Distributions of initial landing positions in the test words. Across-participants probability density functions (bandwidth: 1 letter or 0.25°; Gaussian Kernel) of initial landing positions in short and long test words (3–4 letters and 7–8 letters in left and right panels respectively), for different saccadic launch-site distances (in letters relative to the space in front of the test words), binned in two-letter intervals (from upper to lower panels: [0,-2[, [-2,-4[, [-4,-6[, [-6,-8[, referred to as -1,-3,-5 and -7 respectively), and separately for the two most extreme categories of word frequencies (A) and of word predictabilities (B), when these were grouped respectively into four bins (see Materials and Methods). Light-grey rectangle areas represent the horizontal extent of the words.

In Fig 7A and 7B, the distributions of initial landing positions in the test words were represented for a subset of word lengths and saccadic launch-site distances, separately for high- vs. low-frequency and high-vs. low-predictability test words, respectively, but across participants. High- and low-frequency categories, as well as high- and low-predictability categories, were defined after grouping words into four bins; they corresponded to the first and the fourth bin respectively (see Materials and Methods). These distributions first revealed a clear launch-site effect, in accordance with McConkie et al.'s [22] original findings: As saccades’ launch site laid further to the left of the test words (from upper to lower panels), landing-site distributions shifted accordingly, thus moving from the very-end towards the very-beginning of short words (left two panels), and from a position to the right of the words’ center to the words’ beginning in the case of long words (right two panels). Also in line with previous findings, landing-site distributions showed very little variations with the frequency or the predictability of the test words. Still, for long, 7- and 8-letter, test words, landing-site distributions tended to peak slightly closer to the words’ end with increasing frequency (Fig 7A), and to some extent also with increasing predictability (Fig 7B), though mainly in close launch-site cases (> -6 and -4 letters respectively), thus when the test words could benefit from peripheral preview. This is in accordance with the non-word-based view (see also Fig 1), and opposite to the prediction made by word-based models, that language-related effects on within-word landing positions should only occur towards the tails of the distributions. For shorter, 3- and 4-letter, test words, the major part of the distributions associated with high-frequency words tended to lay underneath that for low-frequency words, at least in close launch-site conditions, thus suggesting also a rightward shift. However, the shift likely took place beyond the word boundaries (not plotted here), thus yielding, in the case of short test words, word-frequency and word-predictability effects on the likelihood of word skipping, as shown above, but not on within-word initial landing sites.

LMM of initial landing positions in the test words shed further light on these trends. The model’s fixed effects, summarized in Table 6, first revealed that the eye initially fixated a position slightly to the left of the words' center (intercept estimate: -0.56344) when all variables were at their reference, mean, value, and words were about 6 letters long. As further indicated by the negative slope estimate for the effect of word length (-0.20296), this leftward bias increased as the test words became longer (see also [121]). Furthermore, saccades landed closer to the beginning of the test words as they were launched from further away; the slope estimate for the effect of launch site (0.43429) indicated that for every 1-letter increment of the launch-site distance from the space in front of the test words, landing positions shifted on average by slightly less than half a letter towards the words’ beginning. The launch-site effect mildly increased as the test words became longer, as suggested by the significant interaction between launch site and word length (estimate: 0.04709), thus in contrast with McConkie et al.'s [22] original report of an invariant (0.49) linear relationship between word-center-based launch site and landing site. However, this was not due to launch site being here expressed relative to the space in front of the words. Indeed, LMM with launch-site distance expressed relative to the center of words, and for words of either 3–11 letters or 4–8 letters as in McConkie et al.’s study, also yielded significant interactions between launch site and word length (estimates: 0.05867 and 0.06472 respectively; see S6 Table). Note though that the effect of word length was no longer significant, as in their study (estimate: -0.00153, *t* = -0.07228 for 3- to 11-letter words; estimate: 0.01233; *t* = 0.47741 for 4- to 8-letter words).

**Table 6:** Fixed effects of optimal LMM for initial landing positions in the test words.

	Estimate	Std. Error	t value
**(Intercept)**	-0.56344	0.11509	-4.89566
**FREQ**	0.02252	0.01820	1.23699
**PRED**	0.01517	0.01128	1.34415
**LENGTH**	-0.20296	0.02242	-9.05257
**LAUNCH**	0.43429	0.02497	17.39223
**FREQ:LENGTH**	0.02822	0.00931	3.03042
**FREQ:LAUNCH**	0.00907	0.00575	1.57741
**PRED:LENGTH**	0.01479	0.00635	2.32793
**LENGTH:LAUNCH**	0.04709	0.00517	9.10263
**FREQ:LENGTH:LAUNCH**	0.00517	0.00298	1.73603

Initial eye landing positions were expressed in letters relative to the center of the test words. The fixed structure included the effects of word length (“LENGTH”; 3–11 letters), word frequency (“FREQ”; between -1.97 and 7.16 log units), word predictability (“PRED”; between -2.60 and 2.60 logit units), and saccadic launch-site distance (“LAUNCH”; between -8.00 and -0.002 letters from the space in front of the test words), as well as the two-way interaction between word predictability and word length, the three-way interaction between word frequency, word length and launch site and all corresponding two-way interactions; the random structure included a random intercept by participant and sentence pair, as well as by-participant random effects of word length, word predictability and saccadic launch-site distance (see S1 Table). The intercept estimate gives the initial landing position when all variables were at their reference, mean, value (Word Length: 6.20 letters; Launch Site: -4.39 letters; Word Frequency: 2.91 log units; Word Predictability: -0.97 logit units). Colon stands for interaction. See S5 Table for the corresponding minimalist optimal GLMM.

**Fig 8:**
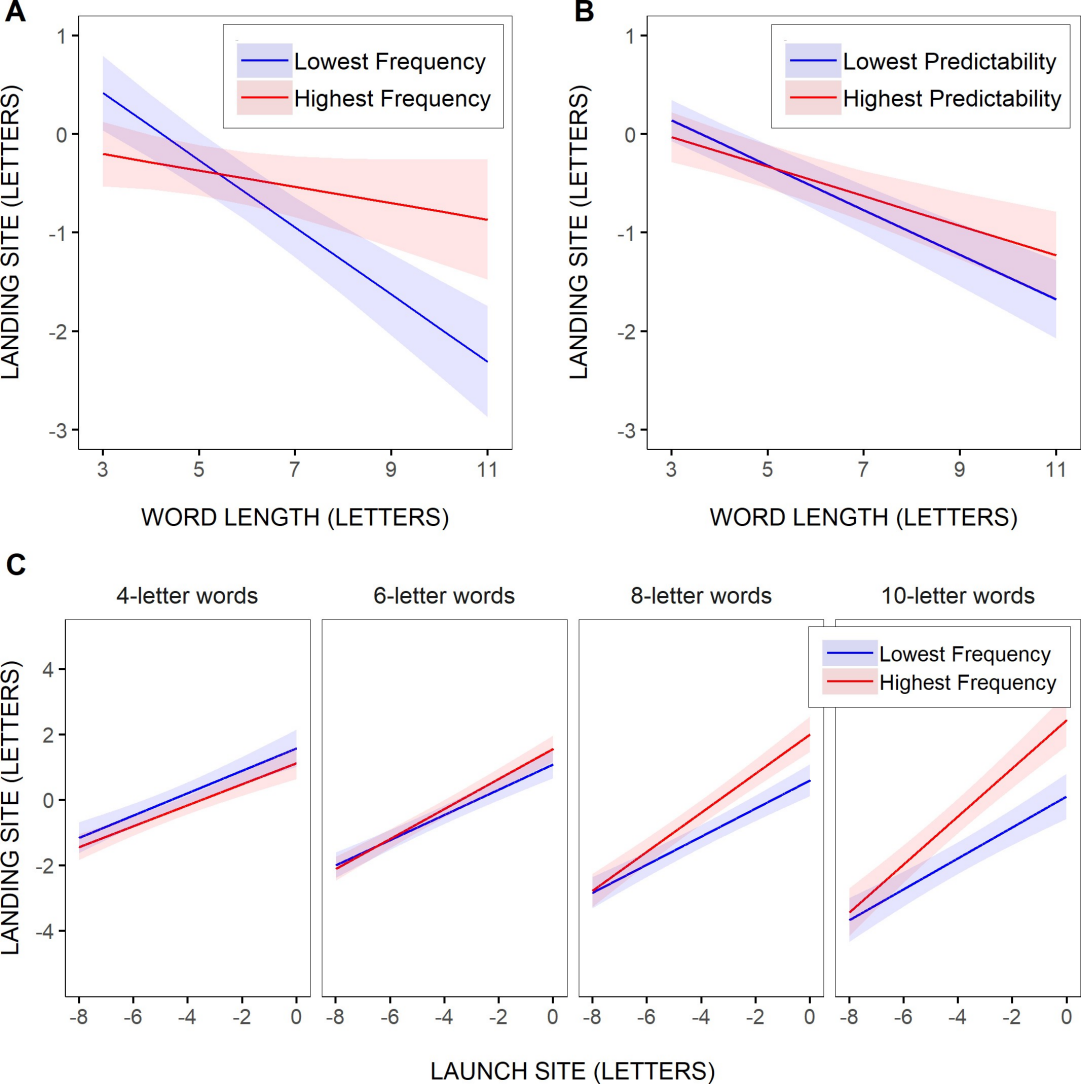
Estimated initial landing positions in the test words. Partial effects (with 95% confidence intervals) computed from LMM parameters (Table 6), representing initial landing positions in the test words as a function of word length (in letters; A-B), and for 4-,6-,8-, and 10-letter test words as a function of saccadic launch-site distance (in letters relative to the space in front of the test words; C), separately for the two most extreme word-frequency values across all test words (-1.97 vs. 7.16 log units; A,C), and the two most extreme word-predictability values across all test words (-2.60 vs. 2.60 logit units; B).

More critical for a test of word-based models, was whether linguistic factors would significantly influence within-word landing positions. As shown in Table 6, neither the frequency nor the predictability of the test words had a significant effect (estimate: 0.02252, *t* = 1.23699, and estimate: 0.01517, *t* = 1.34415). Still, there were significant interactions between word frequency and word length (estimate: 0.02822), and word predictability and word length (estimate: 0.01479). As illustrated in Fig 8A and 8B, where the model’s predictions were represented, using the two most extreme word-frequency and word-predictability values across all test words, saccades landed further into more frequent, and to a lesser extent more predictable, test words, but progressively more as word length increased, and actually only when the words were longer than about 6–7 letters. The marginally significant interaction between word frequency, word length and saccadic launch-site distance (estimate: 0.00517, *t* = 1.73603), suggested in addition that the tendency for saccades to land further into more frequent words, tended to become greater with decreasing launch-site distance, and even more so as word length increased (see Fig 8C). Yet, however consistent the effects of word frequency and word predictability were, they remained much smaller than the effects of launch-site distance and word length, and they were actually smaller than represented in Fig 8A and 8C, given in particular the much smaller range of word frequencies with increasing word length.

In sum, initial eye landing positions in the test words were primarily a function of the words’ length and eccentricity. However, they also varied with the words’ frequency and predictability, though only when the words were long enough for the frequency-related shift in landing-site distributions to take place within the word boundaries (see Figs 1, 7A and 7B), and also essentially when saccades’ launch-site distance was small enough so that the words could benefit from peripheral preview. These effects yet remained smaller than the effects of word length and launch site, as reported above for the likelihood of word skipping. This suggests, in contradiction with word-based models, that language-related variables nearly equally influence the likelihood of word skipping and within-word initial landing positions, and that one or the other occurs depending on the word’s length.

### Within-word initial landing positions across all words in the sentences

In the above, test-word, analyses, we reported tiny effects of linguistic variables on initial landing positions in longer and less eccentric words. To ensure that this pattern was not due to the specific (linguistic) properties of the test words, and that it could be observed at a larger scale and with a greater number of observations, we conducted again the same analyses, but using this time all words in the sentences that responded to the above-selection criteria (see Materials and Methods).

**Fig 9:**
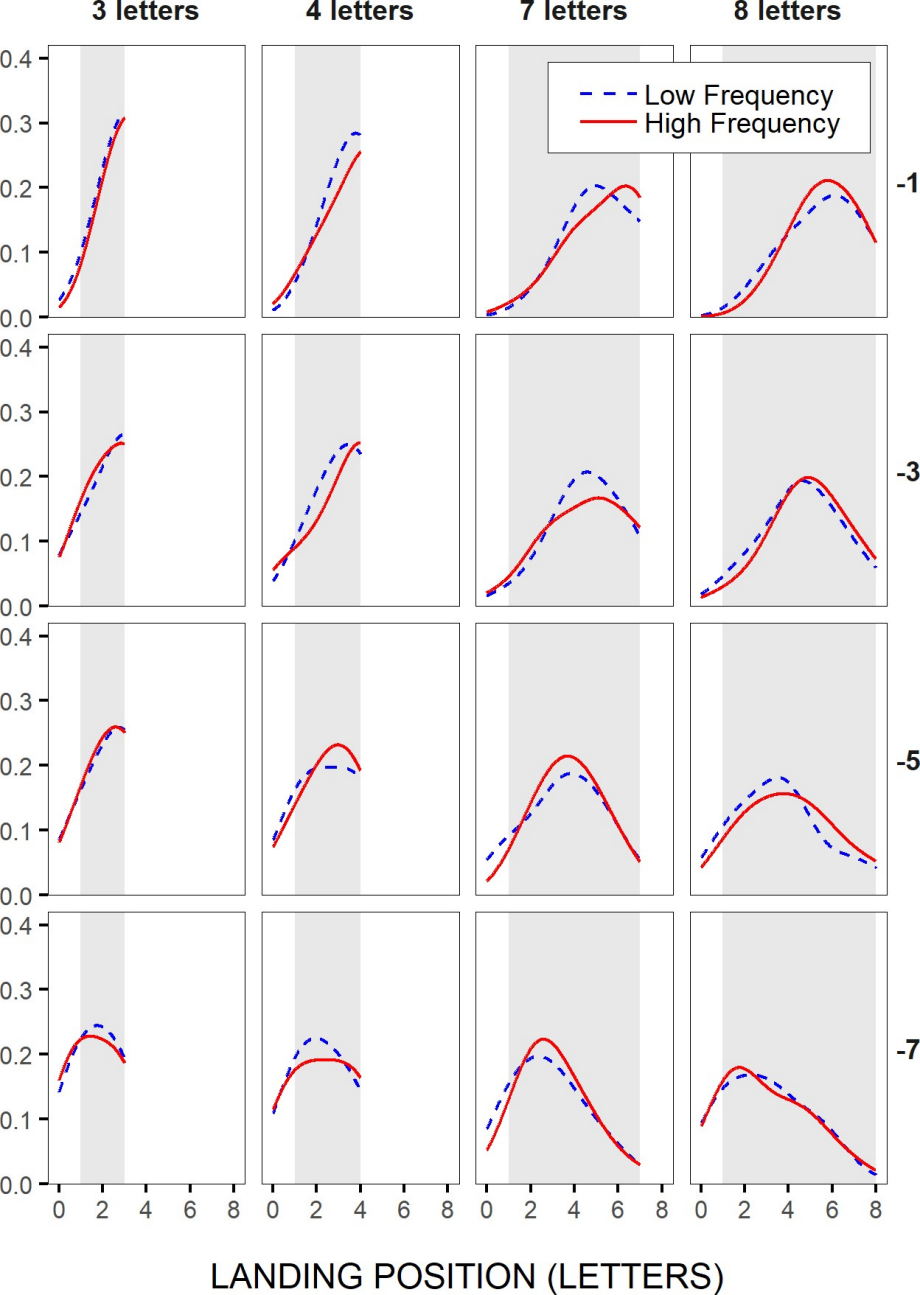
Within-word landing-position distributions. Across-participants probability density functions (bandwidth: 1 letter or 0.25°; Gaussian Kernel) of initial landing positions in short and long words (3–4 letters and 7–8 letters in left and right panels respectively), and for different saccadic launch-site distances binned in one-letter intervals (from upper to lower panels: -1,-3,-5, and -7 letters respectively relative to the space in front of the words), separately for the two most extreme categories of word frequencies grouped into four bins (see Materials and Methods). Light-grey rectangle areas represent the horizontal extent of the words.

In Fig 9, the distributions of initial landing positions in short and long words (3–4 letters and 7–8 letters respectively), were plotted separately for different saccadic launch-site distances to the space in front of the words, and for two categories of word frequencies (low vs. high; see Figure Legend). These again showed, in line with the well-established launch-site effect, that landing-site distributions shifted towards the words’ end as saccades were launched from closer to the words’ beginning [22]. Most importantly, in near and intermediate launch-site cases (> -7 letters), that favored peripheral preview, there was an overall tendency for the distributions to peak slightly further into high- compared to low-frequency words of 7 and 8 letters, thus when the distributions peaked near the center of words. In shorter (3- and 4-letter) words, to the contrary, there was no clear word-frequency related shift in landing-position distributions, at least within the word boundaries. Thus, the pattern reported above for the test words replicated here.

The fixed effects of the corresponding LMM are presented in Table 7. The emerging pattern was consistent with that observed for initial landing positions in the test words. Saccades initially landed at a position slightly to the left of the words' center when all variables were at their reference (mean) value, and words were about 6 letters long (intercept estimate: -0.62161). However, they landed closer to the end of shorter and less eccentric words, as suggested by the negative and positive slope estimates for the effects of word length and saccadic launch-site distance (-0.22438 and 0.35929 respectively). The interaction between launch-site distance and word length was significant (estimate: 0.04516), indicating that the launch-site effect became slightly greater with increasing word length. This was again unrelated to launch-site distance being expressed relative to the space in front of the words; when within-word landing positions were re-analyzed as a function of word length and word-center-based launch-site distance, the interaction remained significant, while the effect of word length was now only marginally significant (see S8 Table).

**Table 7:** Fixed effects of optimal LMM for within-word initial landing positions.

	Estimate	Std. Error	t value
**(Intercept)**	-0.62161	0.08112	-7.66287
**FREQ**	0.01986	0.01013	1.96007
**LENGTH**	-0.22438	0.01647	-13.62216
**LAUNCH**	0.35929	0.01672	21.48509
**FREQ:LENGTH**	0.01695	0.00246	6.88573
**FREQ:LAUNCH**	-0.00191	0.00210	-0.90968
**LENGTH:LAUNCH**	0.04516	0.00260	17.35898
**FREQ:LENGTH:LAUNCH**	0.00414	0.00076	5.41330

This analysis was conducted across all words in the sentences that responded to our selection criteria (see Materials and Methods). Within-word initial landing positions were expressed in letters relative to the center of words. The fixed structure included effects of word length (“LENGTH”; 3–11 letters), word frequency (“FREQ”; between -2.66 and 9.59 log units), and saccadic launch-site distance (“LAUNCH”; between -9.99 and -0.001 letters from the space in front of the words), as well as all interactions; the random structure included a random intercept by participant, sentence pair, and word, as well as by-participant random effects of word frequency, word length and saccadic launch-site distance (see S1 Table). The intercept estimate gives the initial landing position when all variables were at their reference, mean, value (Word Length: 5.94 letters; Launch Site: -4.86 letters; Word Frequency: 4.11 log units). Colon stands for interaction. See S7 Table for the corresponding minimalist optimal GLMM.

**Fig 10:**
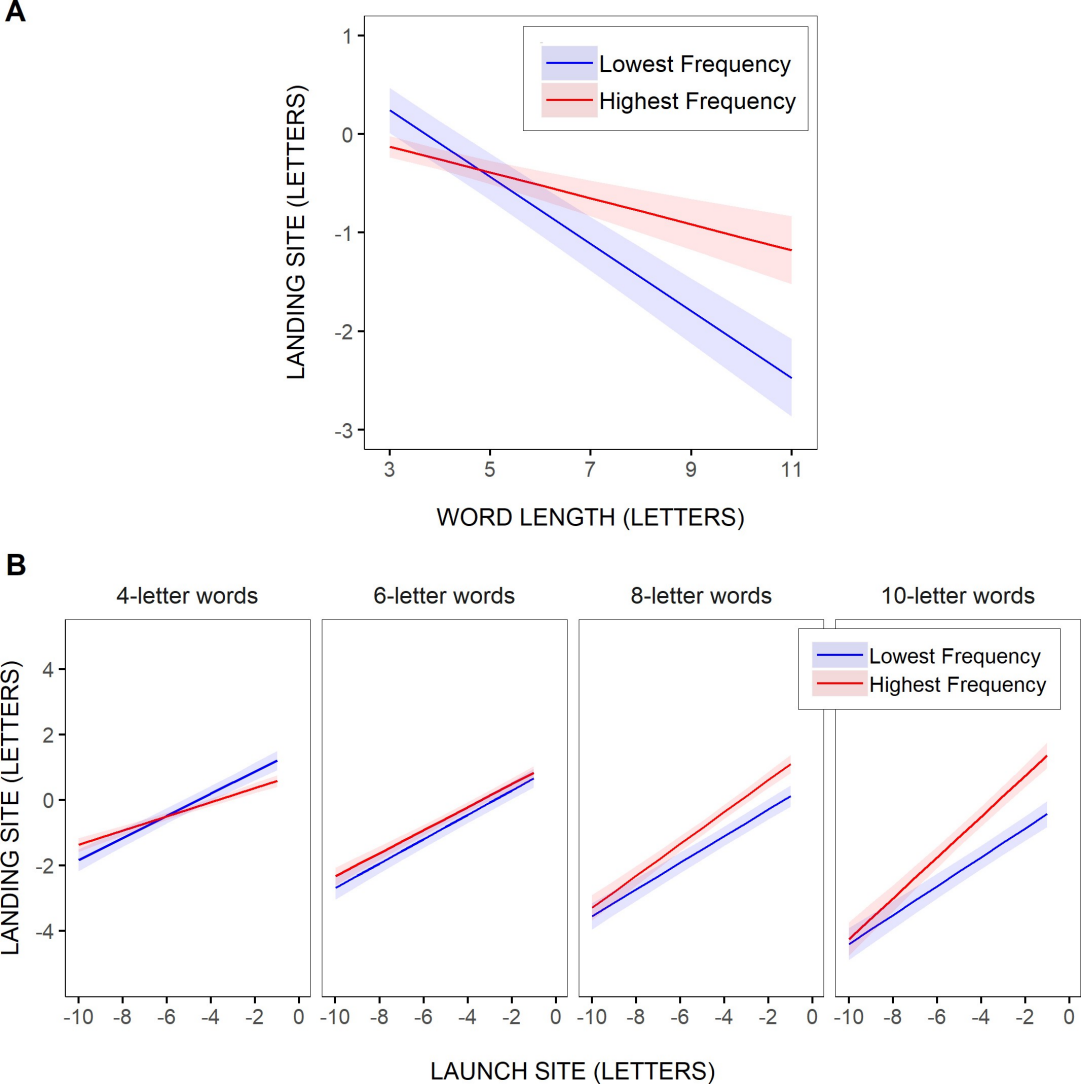
Estimated within-word landing positions. Partial effects (with 95% confidence intervals) computed from LMM parameters (Table 7), representing within-word initial landing positions for all words in the sentences as a function of word length (in letters; A), and for 4-, 6-, 8-, and 10-letter words as a function of launch-site distance (in letters relative to the space in front of the words; B), separately for the two most extreme word-frequency values across all selected words regardless of their length (-2.66 and 9.59 log units respectively; B).

Most importantly, although there was now a marginally significant effect of word frequency on within-word initial landing positions (estimate: 0.01986; *t* = 1.96007), both the interaction between word frequency and word length and the interaction between word frequency, word length and launch-site distance were again significant (estimate: 0.01695 and 0.00414 respectively). The positive slope estimates indicated a tendency for saccades to land slightly closer to the words’ end as their frequency increased, with this tendency becoming greater in longer words, and even more so as saccades were launched from closer to the words' beginning. This is illustrated in Fig 10A and 10B, where the model’s predictions for the effects of word length and launch-site distance were represented for the two most extreme word-frequency values across all selected words. From this figure, it is again quite clear that the effect of word frequency remained relatively small in comparison with the effects of word length and launch site. This was only about half of the effect of launch-site distance in the most optimal conditions (longest word and smallest launch-site distance), and actually much less since the range of word frequencies for a given word length was less than the range of word frequencies across all words. Still, the fact that there was an effect of word frequency at least in long words does suggest that within-word landing positions, just as word-skipping likelihood, are slightly modulated by language-related variables.

In sum, despite word length and launch site were strong predictors of initial landing positions in words, word frequency did also slightly, though significantly, contribute. Importantly, its impact was greater when landing positions were on average away from the word boundaries, as in the case of long words, and when the saccades were launched from close enough to the words’ beginning so that the words could benefit from peripheral preview. This is clearly in contradiction with predictions from word-based models, but in line with the assumption that saccades are overall slightly modulated by linguistic processing, regardless of word boundaries.

### Overall landing positions (regardless of word boundaries)

In the above analyses, we found that word frequency, and to some extent word predictability, not only influenced the likelihood of word skipping, but also within-word landing positions, at least for some word lengths and/or saccadic launch-site distances. Critically, while word frequency had a greater impact on the likelihood of skipping shorter words, it influenced almost exclusively saccades’ initial landing positions in long words. These findings, in contradiction with the predictions from word-based models, provided a first set of evidence for the hypothesis that ongoing peripheral word-identification processes overall modulate where the eye moves, regardless of word boundaries.

The non-word-based view makes yet another, more direct, prediction. It predicts that saccades should land further on the line of text when the word immediately to the right of fixation (N+1) is easier to process, and even more so in optimal peripheral preview conditions, that is when the word is shorter and less eccentric. To test this prediction, we thus re-analyzed the data, but measuring this time the landing positions of all saccades launched from a given word (N), regardless of the word they landed on, as a function of the properties of Word N+1 and saccades’ launch site distance to the space in front of Word N+1. These overall landing-position analyses, unlike the above analyses, did not imply word-based truncation of landing-site distributions (see also [39, 122, 123]). Saccades between 0 and n words in length were assumed to belong, at least by default, to the same population, thus allowing a more objective/neutral test of word-based vs. non-word based hypotheses, while avoiding limitations due to floor/ceiling effects as in the above word-skipping rate and within-word landing-position analyses.

These overall saccadic landing-position analyses were conducted across all words in the sentences. Indeed, given the wider range of possible landing positions, in comparison with within-word landing positions, the n was too low for these analyses to be conducted over the test words only. The same selections as for within-word landing-position analyses were applied, except that the fixation of interest was part of the first eye pass on a word, and hence not necessarily the first fixation on a word: this corresponded either to a refixation of Word N or the first fixation on one of the following words (Word N+1, N+2 …). The critical word, N+1, was between 3 and 11 letters, not the first or last word on the line, not preceded or followed by punctuation, and not a compound word. In addition, the fixation of interest was within a window of -10 to 20 letters around the center of Word N+1.

Assuming non-word-based eye-movement guidance, we expected that overall landing-site distributions would shift further towards the end of the line for high- compared to low-frequency N+1 Words, though more largely as the words were shorter and less eccentric. In contrast, word-based models, predicted at least bimodal distributions, centered respectively on Words N+1 and N+2, with a smaller peak associated with high- compared to low-frequency N+1 Words, but no word-frequency related shift in landing positions. As further detailed below, the data were inconsistent with these latter predictions, arguing instead for non-word-based eye-movement guidance.

**Fig 11:**
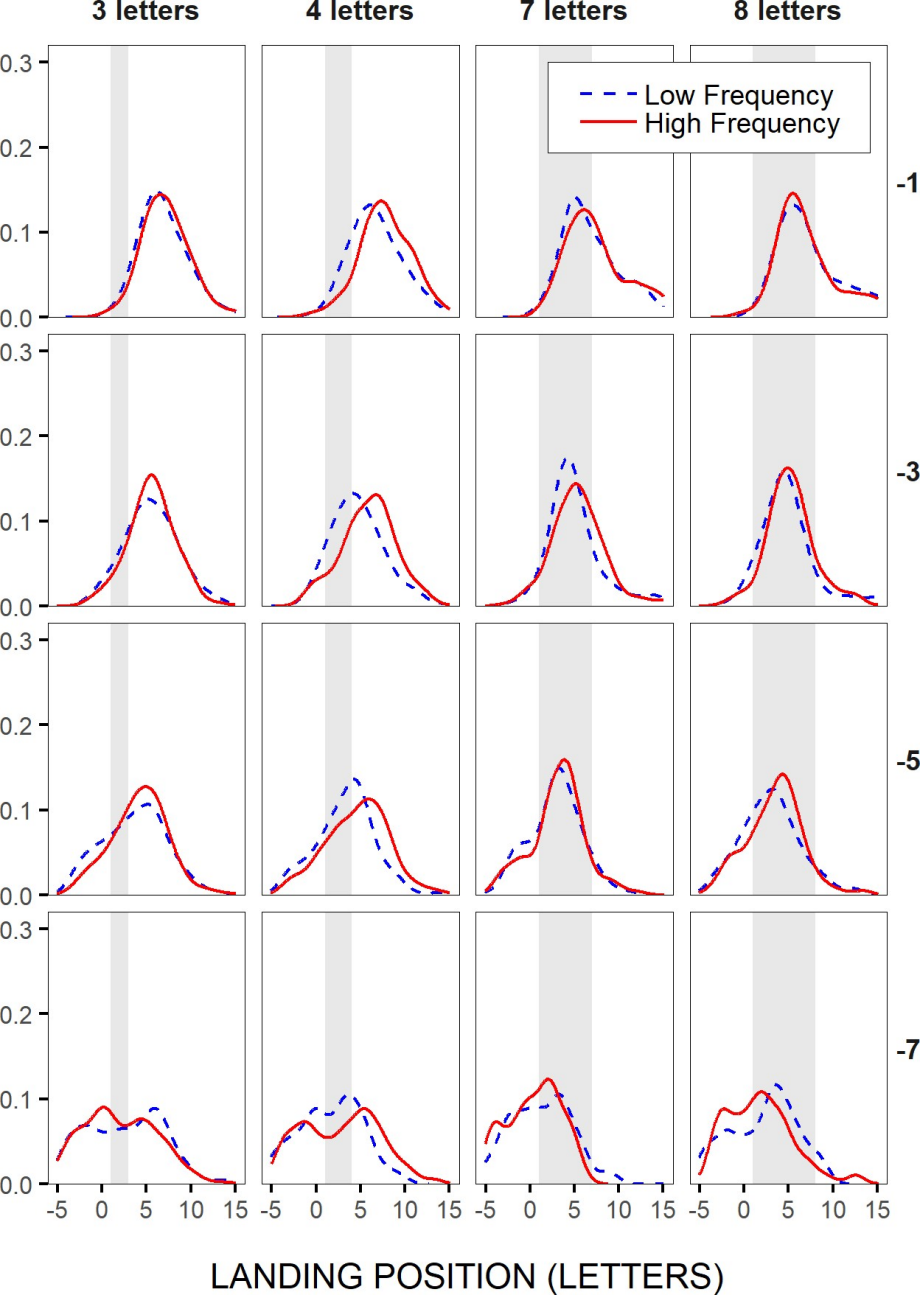
Overall landing-position distributions. Across-participants probability density functions (bandwidth: 1 letter or 0.25°; Gaussian Kernel) of all saccades’ landing positions regardless of word boundaries (or overall landing positions), expressed in letters relative to the beginning of Word N+1 (i.e., the word immediately to the right of the word (N) from which the saccade was launched), with positive values corresponding to landing positions on this word or beyond it, and negative values corresponding to landing positions in front of the word, and hence refixations of Word N. Distributions were plotted, using all words in the sentences that responded to our selection criteria (see Text), separately for short and long N+1 words (3–4 and 7–8 letters in left and right panels respectively), different saccadic launch-site distances (in letters relative to the space in front of Word N+1), binned in two-letter intervals (from upper to lower panels: [0,-2[, [-2,-4[, [-4,-6[, [-6,-8[, referred to as -1,-3,-5 and -7 respectively), and for high vs. low-frequency N+1 words (i.e., the words that fell respectively within the two most extreme categories of word frequencies when grouped into three bins; see Materials and Methods). Light-grey rectangle areas represent the horizontal extent of the words.

In Fig 11, overall landing-site distributions across all words in the sentences were plotted for two categories of word frequencies (low vs. high; see Figure Legend), separately for short (3- and 4-letter) and long (7- and 8-letter) words and for different saccadic launch-site distances (in 2-letter bins). The distributions were for the great majority unimodal. There was only a tendency for the right tail of landing-site distributions to be elongated in the case of longer and less eccentric words (upper right panels), as well as a tendency for somewhat bimodal distributions at the largest launch sites (lower panels), although it is hard to tell whether the latter was due to a lack of data or within-word refixations forming a separate population. In any case, there was clearly no evidence for the distributions to exhibit two distinct modes, with one centered on Word N+1, and the other centered on Word N+2. Actually, most saccades landed beyond the end of very short (3- and 4-letter) N+1 words, and within the boundaries of long (7- and 8-letter) N+1 words, with the exact landing position relative to the beginning of N+1 words being primarily a function of the saccades’ launch-site distance to the space in front of the words as well as the words’ length. As saccadic launch-site distance increased, the distributions shifted leftward, peaking closer to the end/center of short words, and the very-beginning of long words (or in front of it). Moreover, as word length increased, the distributions peaked slightly closer to the words’ beginning. Most importantly, there was a slight, though quite consistent, rightward shift in landing-site distributions with increasing word frequency; this indicated that saccades tended to land slightly further as N+1 words were more frequent, though to greater extents when the words were shorter (and in particular 4 letters long) and not too far out in the periphery (< 7 letters). As a result, word frequency nearly exclusively influenced the likelihood of word skipping in the case of short words, while mainly affecting within-word landing positions in the case of long words, in line with the above analyses.

An LMM was fitted to overall saccadic landing positions, as measured from the beginning of N+1 Words, using the same cut-off selections for word length and launch-site distance as in within-word landing position analyses. As shown in Table 8, where the model’s fixed effects were reported, saccades landed 1.7 letters away from the beginning of N+1 Words, when all variables were at their reference, mean, value, and words were about 5 letters long. The positive slope estimate for the effect of launch-site distance (0.94341), indicated that landing positions shifted by only a bit less than one letter for every one-letter increment of the launch-site distance, thus suggesting that the launch-site effect more than doubled its size when all saccades’ landing positions, instead of only within-word landing positions, were considered for analysis (see Table 7 for comparison). Note that this was not a result of saccades’ landing positions being measured relative to the beginning of N+1 Words. When data were re-analyzed using word-center-based launch sites and landing sites, a similar slope was obtained (estimate: 0.93986; S9 Table). This first result confirms that the launch-site effect extends well beyond the word boundaries, while showing that its slope varies with how data are analyzed ([21, 39], see also [30]). In the discussion below, we will see that this is also inconsistent with predictions from word-based models.

**Table 8:** Fixed effects of optimal LMM for overall landing positions.

	Estimate	Std. Error	t value
**(Intercept)**	1.73141	0.22040	7.85565
**FREQ**	0.10661	0.01754	6.07823
**LENGTH**	-0.43359	0.02456	-17.65153
**LAUNCH**	0.94341	0.02312	40.80692
**FREQ:LENGTH**	-0.02027	0.00517	-3.91887

Were considered for analysis, the landing positions of all saccades regardless of word boundaries; these were expressed in letters relative to the center of Word N+1, that is the word immediately to the right of the word (N) from which the saccade was launched. Word N+1 was not necessarily a test word (see Text). The fixed structure included effects of word (N+1) length (“LENGTH”; 3–11 letters), word (N+1) frequency (“FREQ”; between -2.66 and 9.59 log units), and saccadic launch-site distance (“LAUNCH”; between -9.99 and -0.001 letters from the space in front of Word N+1), as well as the interaction between word frequency and word length; the random structure included a random intercept by participant, sentence pair, and word, as well as by-participant random effects of word frequency, word length and saccadic launch-site distance (see S1 Table). The intercept estimate gives saccades’ landing position when all variables were at their reference, mean, value (Word Length: 5.06 letters; Launch Site: -3.27 letters; Word Frequency: 5.44 log units). Colon stands for interaction. The corresponding minimalist optimal LMM was identical.

**Fig 12:**
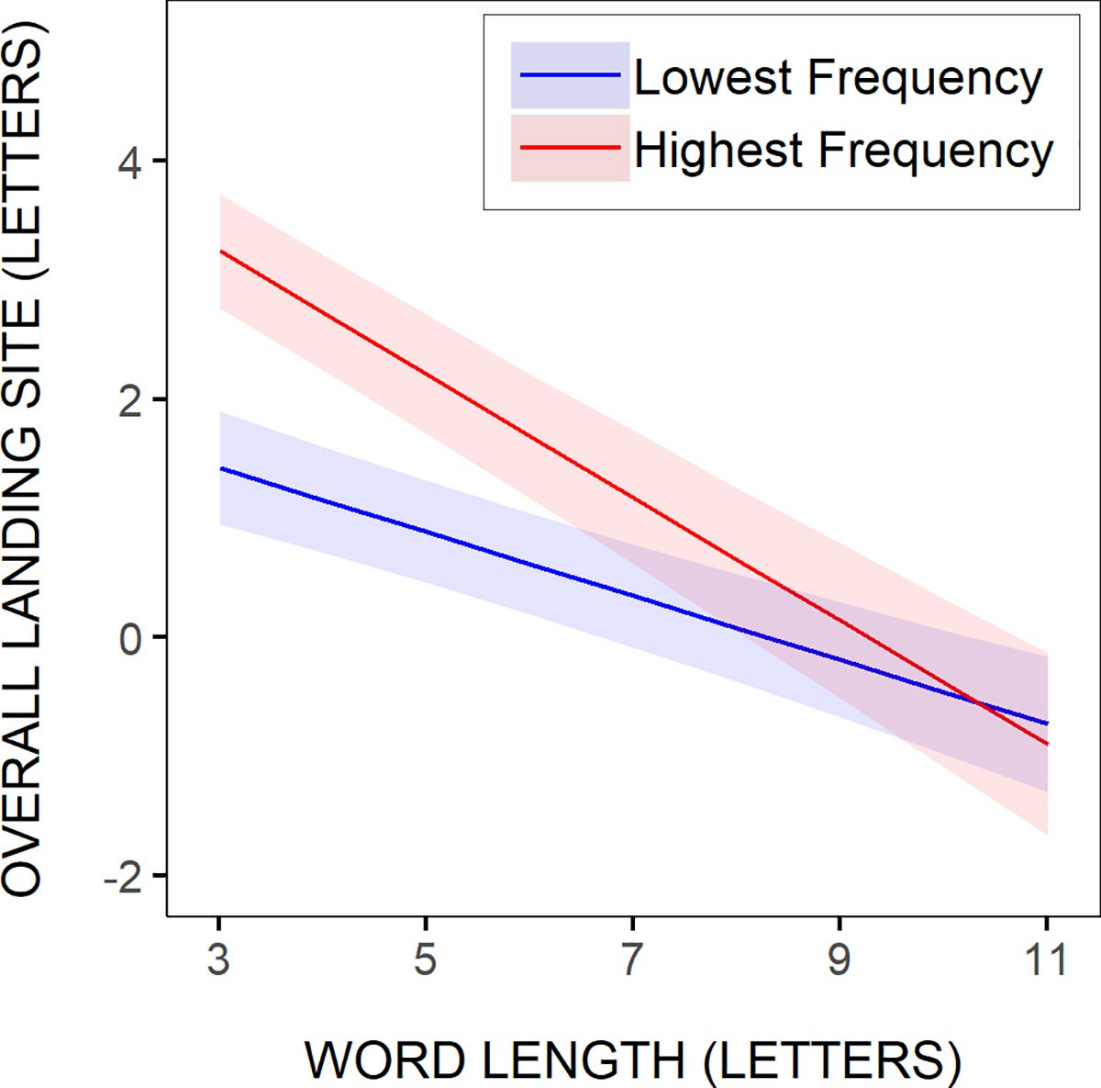
Estimated overall landing positions. Partial effects (with 95% confidence intervals) computed from LMM parameters (Table 8), representing saccades’ overall landing positions for all words in the sentences as a function of word length (in letters), separately for the two most extreme word-frequency values across all selected words (-2.66 and 9.59 log units respectively).

The model’s fixed effects additionally revealed a significant effect of word length, suggesting that saccades landed closer to the beginning of longer N+1 Words (estimate: -0.43359). More critically, there was a significant effect of word frequency (estimate: 0.10661), as well as a significant interaction between word frequency and word length (estimate: -0.02027). This indicated that saccades landed further away with increasing word frequency, and even more so as words were shorter and hence more greatly benefited from peripheral preview. As shown in Fig 12, where the model’s predictions were represented for the two most extreme word-frequency values across all selected words, there was a clear word-frequency effect for short, 3- and 4-letter, words. Given that these words were most often skipped, this indicates that even word-skipping saccades landed at different locations on the line depending on the words’ frequency. The additional fact that the word-frequency effect extended to longer words, that were most often fixated, confirmed the above-reported effect for within-word landing positions.

Thus, contrary to the predictions made by word-based models, but in line with the non-word-based view, linguistic variables did influence where the eye moved on the line of text, regardless of word boundaries. Their effects were smaller in longer words, that less largely benefited from peripheral preview, and much smaller compared to the effects of saccadic launch-site distance and word length, therefore suggesting that linguistic variables modulated only occasionally the length of default forward saccades, as determined based on low-level visuo-motor mechanisms.

## Discussion

To test the general hypothesis that eye movements during reading are purposely guided from one word to another word based on the (expected) needs of ongoing word-identification processing, we re-examined the long-studied influence of language-related variables on forward eye-movement behavior, but using linear-mixed-effect modeling applied to a large and well-controlled sentence-reading data set. We found that the words’ frequency of occurrence in the language, and their predictability from the sentence context (in the case of test words), only mildly influenced where the eye moved next, in comparison with the words’ length and the saccades’ launch-site distance to the beginning of words. Nevertheless, frequency and predictability affected not only the likelihood of word skipping, but also within-word landing positions, all depending on the words’ length and eccentricity. Words that were shorter (3–5 letters long), and also closer to the saccade’s launch site, were more often skipped, and even more so as their frequency, and/or their predictability increased. However, as word length increased, the likelihood of word skipping became both smaller and less strongly affected by word frequency/predictability, while within-word landing positions, closer to the words’ center, started showing variations with frequency and predictability. As suggested in further analyses, these effects came from an overall slight shift of saccades’ landing positions towards the end of the line of text, with increasing easiness of Word N+1. In the next sections, we explain how these novel findings contradict the predominant top-down word-based account of eye-movement guidance during reading. We then argue, in line with Vitu’s [5, 6] bottom-up, non-word-based, CoG hypothesis, that saccades drive the eye forward along the lines of text regardless of word boundaries, primarily as a result of low-level, non-word-based, spatial-integration mechanisms, and only exceptionally based on ongoing language-related processes.

### Evidence against top-down word-based eye-movement guidance

The general hypothesis in top-down word-based models, that the metrical properties of saccadic eye movements during reading result from a compromise between a saccade-targeting strategy towards the center of peripherally selected target word(-object)s and SRE ([1–4, 19], see also [14–16, 20]), relies on two main arguments. The first relates to the many empirical findings showing that the words that are skipped are more easily processed in peripheral vision: they are not only shorter [9, 20] and less eccentric [44, 45], but they are also visible (in opposition to being masked in peripheral vision [46–49]), highly frequent [50–56], and/or highly predictable [20, 48, 50, 55, 58–65]. The second argument relates to the well-established fact that within-word landing positions systematically vary with saccades’ launch-site distance to the center of words [20, 22, 30, 31], but often fail to show clear and significant variations with the words’ linguistic properties (for reviews see [6, 21, 56, 67]).

In line with these findings, and hence word-based models, our results first confirmed that the likelihood of word skipping varies with word length and saccadic launch-site distance, as well as the frequency, and to a lesser extent the predictability, of words. However, the fact that frequency and predictability effects, which occurred mainly in shorter and less eccentric words, remained much smaller than the effects of word length and saccadic launch-site distance (see also [44, 54, 66, 71], but see [72]) may represent a challenge for top-down, word-based (and non-word-based), models, and E-Z Reader and SWIFT [1, 2] in particular. Both models can predict a reduction in word-skipping rate with increasing word length (and eccentricity). Still, given the models’ underlying assumption that selection of a saccade-target word relies on ongoing word-identification processing weighted by letter eccentricity, it remains undetermined whether the models’ predicted effect of word length is lexical or visual in nature, and hence whether it would be much greater than the effect of lexical variables. As word length is negatively correlated with word frequency, this could, at least partly, be an effect of word frequency (and vice-versa) [124]. The proof is that SWIFT not only requires switching off lexical processing, but also a re-adjustment of the letter-visibility function (and additional assumptions) to predict a quasi-similar length effect during the reading of meaningless (z-transformed) text material ([73]; for E-Z reader applied to z-reading see [125]). GLENMORE makes a distinction between visual and lexical processes: It assumes that early-triggered saccades are guided in a blob-based manner, simply based on ongoing non-lexical visual processing (i.e., visual-acuity function [4, 126]), and that later-triggered saccades reflect lexical, word-based guidance. This model should therefore more easily account for our observation that word length more greatly affected the likelihood of word skipping than linguistic variables. Whether GLENMORE would provide a sensible account for the reduction in skipping rate with increasing letter-string length and saccadic launch-site distance during the reading of meaningless z-transformed texts [73–76], however remains debatable. Since z-letter strings have no linguistic content and are 100% predictable, they should always be skipped, regardless of their length, unless word-skipping behavior reflects hard-wired pre-determined visuo-motor scanning routines that cannot be turned-off in the absence of linguistic content and/or in low uncertainty conditions [11, 12].

Our landing-position findings however provided further and unambiguous arguments against word-(object-)based accounts of eye-movement guidance during reading. Our first observation that within-word landing positions were part of a larger, a-priori unimodal, distribution of saccades’ landing positions, that largely extended outside the word boundaries (see also [39]) is already in contradiction with the prediction made by word-based models, that there should be as many modes as possible target words (minimally the next word, N+1, and the word following it, N+2). Our additional finding that these distributions shifted by about 0.9 letter towards the beginning of Word N+1 (or even in front of it) with every one-letter increment of the saccades’ launch-site distance to Word N+1 (for similar findings during Chinese reading see [21, 53]) simply is inconsistent with the general hypothesis that saccades’ landing positions result from a compromise between a word-center saccade-targeting strategy and SRE. This hypothesis was proposed precisely because it was thought that there is a relatively invariant linear relationship between launch site and landing site, with a typical slope of 0.5, which is just halfway between a slope of 0 that would indicate that the eyes always land at the center of words, and a slope of 1 that would reflect a tendency to make constant eye steps forward [22]. Using the same rationale, the here-observed slope of 0.9 would mean that saccades in our study were mostly driven by SRE, and hence mostly prone to move the eye a constant distance forward. However, this was unlikely the case because saccades’ landing positions were also strongly influenced by the length of peripheral words. Note in addition, that several previous studies showed that the slope of the linear relationship between saccades’ launch sites and within-word landing sites is not invariant, but rather depends on the peripheral visual configuration [39, 127, 128]. Accordingly, but in contradiction with McConkie et al.’s [22] original findings, we found that the effect of launch-site distance on (within-word) landing positions became stronger with increasing word length.

Another strong argument against word-based guidance came from our finding that within-word landing positions, and even more so overall landing positions, were not exclusively influenced by saccadic launch-site distance and word length, but also depended on the words’ linguistic properties. Just like word-skipping rate slightly increased with increasing word frequency and to some extent also word predictability, within-word landing positions mildly shifted towards the end of more frequent and more predictable words. The fact that these language-related effects on within-word landing positions intervened mainly in long words, while the same effects on word-skipping likelihood occurred mainly in short words, is not surprising when considering that analyses of within-word landing positions rely on truncated landing-site distributions. Since saccades’ overall landing-position distributions peaked towards the center of long words, but near the very-end of short words or even beyond it, they could yield effects of linguistic factors on within-word landing positions mostly in long words (see Figs 1 and 11). The fact yet that less information can be gathered from long words, in comparison with short words, in the periphery, combined with the slowness of language-related processes [42, 43], explains why these effects remained tiny. It also accounts for the fact that many previous studies failed to observe effects of word frequency or word predictability on initial fixation locations in words during the reading of alphabetic languages [56, 61, 62, 69, 70, 79–84]. Variations in within-word landing positions with the words’ orthographic and/or morphological properties were however largely reported ([56, 82, 83, 85, 88–94, 96–103], but see [52, 70, 95, 104–105]). Most importantly, word-frequency and word-predictability effects were also found in a couple of studies and most often in conditions similar to ours, that is mainly in long test words (> = 7 letters on average [55, 56, 85–86]; but for an effect in shorter words see [87]), just as the effects of orthography and morphology (see in particular [56]). Moreover, these effects were much smaller than the effects of word length and saccadic launch-site distance, as in our study. Thus, the fact that Rayner et al.’s [69] data revealed only a tiny, though non-significant, effect of word predictability on within-word landing positions at close-launch sites, was likely due to their words being too short (5–6 letters): as the distributions peaked very near to the end of words (at least in their Experiment 2), the effect mainly took place beyond the word boundaries, being significant only for the likelihood of word skipping.

Interestingly, Liu and colleagues [21, 64] reported very similar findings to ours for the reading of Chinese sentences. They showed that word frequency, word predictability, and peripheral preview significantly modulated saccades’ overall landing positions on the line of text, though much less than saccadic launch-site distance. Still, the former, language-related, effects sometimes yielded effects on within-word landing positions [21, 108], but some other times resulted in variations in word-skipping rate [53, 64]. This was likely because their words, though only two-characters long, were of about the same angular extent (2°) as the smallest words that yielded significant language-related variations in within-word landing positions in our study (8 letters or 2°), and in previous studies (1.98°-4.5°). Indeed, Yen et al. [48] reported a marginally significant effect of word frequency on the likelihood of word skipping, but no effect on within-word landing positions, for 2-character words that subtended only about 1.64°. In contrast, Zhou et al. [106] found an effect of word frequency on within-word landing positions, but no effect on word-skipping likelihood, for words extending about 2.4° (see also [31]). Note that these authors additionally showed an effect of word-boundary ambiguity on within-word landing positions (see also [129], but see [130]), which they interpreted as evidence for flexible saccade-target selection in Chinese reading (i.e., towards the center or the beginning of words, depending on the success of word segmentation). However, since word segmentation inevitably plays a role in word identification, their effect, nearly as small as (previously reported) word-frequency effects, could well be another instance by which ongoing word-identification processes modulate default saccade amplitude.

Finally, our additional finding that word frequency influenced saccades’ landing positions on the line of text even in the case of short and near words, that were most often skipped, further strengthens our non-word-based interpretation of previous word-based results. Assuming that ongoing peripheral word-identification processes only have all-or-none influences on selection of a saccade target word simply cannot lead to the prediction that both skipping and non-skipping saccades would land further away from the beginning of Word N+1 as this becomes easier to process. These findings also suggest that word-based analyses of saccadic behavior can be misleading [39].

Thus, in contradiction with the predominant word-based account of eye-movement guidance, saccades during the reading of alphabetic, as well as un-spaced non-alphabetic, languages, do not seem to rely on segmentation of the text into saccade-target word(-object)s, and where they actually land very unlikely reflects a compromise between a (word-center) targeting strategy and SRE. Rather, where on the line of text (and with respect to word boundaries) the eyes move next would primarily be a function of the peripheral visual configuration on a given eye fixation, as determined by the words’ length and eccentricity. Language-related processes would also intervene, but they would overall modulate saccades’ landing positions regardless of word boundaries, rather than exclusively influencing the likelihood the next word(s) is(are) skipped. Moreover, this would happen essentially when all conditions (word length and eccentricity) are met for an optimal peripheral preview of the word(s), and even more so when the word’s linguistic properties (frequency or predictability) combine to further reduce uncertainty.

### An alternative, bottom-up, non-word-based account of eye-movement guidance

Several models of eye-movement control during reading have already been proposed, that do not involve word-based saccade-targeting processes. The great majority relies on the idea, originally proposed in McConkie’s [36] perceptual-span theory, that readers move their eyes towards the next location on the line, that optimizes the processing of new visual information, given the amount of information acquired from the prior eye fixation (for a review see [67]). Though rapidly abandoned to the profit of top-down word-based eye-movement guidance (for reviews see [6, 11, 68]), this theory was recently revisited to account for Chinese reading [21, 53], as well as reading with a macular scotoma ([17, 34, 35], see also [131]). According to the former, Dynamic Saccade Adjustment (DSA) model, the length of forward saccades would be adjusted continuously based on the amount of peripheral preview, as determined by prior fixation duration, and both the frequency and the visibility of the next word in peripheral vision. On the other hand, according to Mr. Chips, an ideal observer model of reading with a macular scotoma, saccades would be guided towards the next location on the line that minimizes uncertainty on the currently processed word, given both visual acuity and crowding, combined with lexical inferences.

Mr. Chips, and likely also DSA, can simulate sighted readers’ seemingly word-based eye-movement behavior (e.g., the greater likelihood of skipping shorter and easier words). Moreover, both models can account for the launch-site effect, and without making recourse to the greatly debated SRE hypothesis ([27, 28, 39], see also [127, 128, 132–134]); assuming this results from ongoing visual and lexical word-identification processes within the visual/perceptual span, they even (potentially) predict that this effect extends beyond the word boundaries. Still, the models’ processing-based account of the launch-site effect can hardly be reconciled with the fact that previous attempts at showing an influence of the availability of peripheral preview on within-word landing positions either failed [28, 77, 78], or yielded effects that were four to five times smaller than the effect of launch site [22]. In addition, as further developed below, the models’ underlying assumption, as in word-based models, that there is enough time during an eye fixation during reading for visual and language-based top-down selection of a saccade goal is debatable. Thus, the perceptual-(visual-)span account, though non-word-based, does not appear to us as the best possible explanation for eye-movement guidance during reading.

Yang and McConkie [7, 8] were the first to experimentally address the timing issue. Using gaze-contingent display-change manipulations, they showed that inter-word spacing, and even more so word-information content, become available to the saccadic system only late during a fixation (i.e., not before about 175–200 ms and 225–250 ms from fixation onset respectively). On that basis, they proposed the assumption that eye movements during reading are by default purely driven by strategy-based activation, a SRE-like bias to move the eye a constant distance forward (see also [135–136]), and only later visually and linguistically controlled. The authors’ Competition/Interaction (C/I) model relies on this assumption. Although this model is conceptually different from word-based models, it turns out to be as problematic, notably because it makes quite similar predictions for the landing positions of forward saccades. First, given the range of fixation durations during reading, and the fact that 90% of them are longer than 150 ms, this model paradoxically predicts a major role of visual input, at the expense of strategy-based activation [18]. Since visually based guidance is a function of letter-based activation, as weighted by letter eccentricity, letter-distance to the center of words, and word length, this means that saccades would be essentially driven in a word-based manner. Thus, while the model predicts, in line with previous findings, that the eyes should land closer to the words’ beginning as word length and launch-site distance increase [22], it also predicts that the landing-position distributions of forward saccades should be multimodal, with each mode aligned with a possible target word (see Fig 3 in Yang [18]). However, as we have seen above, this is not the case. In addition, given the predominance of visually based guidance, the slope of the linear relationship between saccades’ launch site and landing sites should be no greater than 0.5, and likely less (see above), thus in contradiction with the here-observed slope of about 0.9. On the other hand, the model’s additional assumption that ongoing language processing contributes only through saccadic inhibition cannot lead to predict an overall shift in saccades’ landing-position distributions towards, or even beyond, the end of easier words, as we observed (see also [108]). When a processing difficulty is encountered, the region in the motor map coding for the planned saccade would be inhibited. This should in turn both reduce the propensity to move the eye forward and inflate the likelihood of short-amplitude forward saccades (or within-word refixations), but it should have no effect on the landing positions of large-amplitude forward saccades. Thus, although we cannot reject all assumptions made in the C/I model, this does not seem to propose a sensible and accurate account for where on the line of text the eyes land.

The alternative, center-of-gravity theory, that was originally proposed by Vitu [5, 6, 40, 137], may provide a more appropriate framework to account for the present findings, and possibly also eye-movement guidance in other, non-alphabetic, languages. Unlike word-based models, this incorporates neither selection of a saccade target word(-object), nor segmentation of the text into perceptual word units, to predict where the eyes land when moving forward. On any given eye fixation during reading, each letter on the line of text would be assigned a given level of activity, depending only on its distinctiveness from the background, and hence also its eccentricity, but regardless of the word it belongs to. Where the eyes move next would then directly derive from spatial-integration mechanisms, the same mechanisms that were shown to determine the metrical properties of saccades in simple saccade-targeting tasks [41]. These mechanisms take place in the Superior Colliculus (SC), a midbrain structure that transforms visual input into the spatial code for a saccade [138]. The SC receives afferents from many cortical areas, but also directly from the retina [139]. As spatial coding is distributed over populations of neurons with large and overlapping receptive/movement fields, saccades move the eyes to the location in space that corresponds to the center of gravity of the entire active population [140]. Given the magnification factor, or overrepresentation of space closer to the fovea ([141–142], see also [143]), the eyes therefore land by default towards a fovea-weighted center of gravity of the global peripheral configuration, meaning away from their target when this is displayed simultaneously, with other, proximal (distractor) stimuli ([133]; for a review see [5]). In a similar manner, saccades during reading would move the eye towards a fovea-weighted center of gravity of the global visual configuration formed by letters to the right of fixation, regardless of letter identity and word boundaries [5, 137]. The resulting overall distributions of saccades’ landing positions should therefore be unimodal, and peak either within or beyond the boundaries of the next word on the line (N+1), depending on the word’s length and eccentricity, as we observed. Saccades launched from close to the beginning of Word N+1 would tend to land beyond the end of the word when it is short, and near the end of the word when it is long, being pulled forward by material ahead of Word N+1. Moreover, as saccades are launched from further away, their landing position would progressively shift towards the word’s beginning, thus reproducing the well-known launch-site effect [22].

The center-of-gravity (or global) effect is a quasi-irrepressible oculomotor response, that vanishes only when saccade latency is greatly prolonged [144], and even more so as the visual array is visually more complex ([132, 145]; for a review see [5]). Top-down, language-based, guidance is therefore not impossible, but given its slowness compared to bottom-up, luminance-contrast, guidance (through the direct retino-tectal pathway [139]), it could only intervene punctually to modulate saccades’ landing positions. This would be the case when fixation durations are prolonged, and/or when visual and linguistic variables combine to favor an early access to the word’s representation. Thus, as we observed, the eyes would land slightly further on the line of text as the frequency and/or the predictability of Word N+1 increases, though more greatly when the word is both short and close enough to fall within the limits of the perceptual span for letter identity (< 6 letters [67]). Still, depending on the word’s length and eccentricity, this would either increase the likelihood the word is skipped or take the eyes further towards the end of the word, as we reported.

MASC, a model of Attention in the SC, accounts for eye-movement guidance in a range of perceptual tasks, simply based on saccade-programming principles in the SC, though taking into account many more SC constraints than originally envisaged in Vitu’s CoG theory [146]. As evidenced in a companion paper, its behavior while viewing sentences from the FSC, very much resembled reader’s eye-movement patterns, even despite this being deprived of language-related knowledge and top-down control [147]. Yet, MASC showed some differences with readers, in line with the here-observed tiny linguistic influences, thus comforting our conclusion that eye-movement guidance during reading is primarily a result of low-level, non-word-based, visuo-motor processes, and only subject to one-off language-based modulations. This model, as the present paper, still only dealt with where, but not when, the eyes move during reading. Whether on ongoing word-identification processes beat visuo-motor factors in determining fixation durations, as predicted by top-down models, and in line with several empirical findings, though not all (for reviews see [6, 67]), is another issue that will be addressed in future work.

## Conclusion

In contradiction with the long-standing assumption that saccadic eye-movements during reading are guided in a word-based manner, we have shown that the frequency, and to some extent the predictability, of words affect both the likelihood of word skipping, and where in the words the eyes land, thus overall influencing saccades’ landing positions regardless of word boundaries. Still, these effects were small, and much smaller compared to the effects of word length and saccadic launch-site distance, which remained the best predictors of readers’ eye movement patterns. Altogether these findings argue for the hypothesis that saccade metrics during reading are primarily determined based on low-level visuo-motor mechanisms that require neither word segmentation nor selection of a saccade-target word(-object) in the periphery. Top-down, language-based, modulations of eye-movement behavior would intervene only in specific instances, notably when the visual and lexical properties of the peripheral word(s) combine to allow a fast access to the words’ representation.

## Supporting information

S1 Table Formulas used for optimal (G)LMMs presented in Tables 2–8 and corresponding minimalist optimal (G)LMMs presented in S2–S5 and S7 Tables. When optimal and minimalist optimal (G)LMMs for a given analysis were identical, only the optimal model’s formula was reported. *SKIP* was a binary variable, indicating whether, or not, the (target) word was skipped. *LP* corresponded to within-word landing positions, and *OVLP* corresponded to saccades’ overall landing positions. *SENTENCE* corresponded to Sentence Pair, and *WORD* corresponded to Word Number. See the corresponding tables’ legends for definition of the predictor variables.
(DOCX)

S2 Table Fixed effects of minimalist optimal GLMM (Model 1) for the probability of skipping the test words. The optimal fixed structure included the effect of word length (“LENGTH”; 3–11 letters) and the interaction between word length and word frequency (“FREQ”; 0.20–5.93 log units; the optimal random structure included a random intercept by participant and sentence pair, as well as a random effect of word length by participant (see S1 Table). The model's estimates and standard errors are expressed in logit units. The intercept estimate (logit: -1.59253) indicates that the probability of word skipping was of about 0.17 when all variables were at their reference, mean, value (Word Length: 5.96 letters; Word Frequency: 3.03 log units). Colon stands for interaction.
(DOCX)

S3 Table Fixed effects of minimalist optimal GLMM (Model 2) for the probability of skipping the test words. The optimal fixed structure included the effects of word length (“LENGTH”; 4–8 letters), and saccadic launch-site distance (“LAUNCH”; between -6.00 and -0.002 letters from the space in front of the test words), and the three-way interactions between word frequency, word length and launch-site distance and between word predictability, word length and launch-site distance; the random structure included a random intercept by participant and by sentence pair, as well as by-participant random effects of word length and launch-site distance, but without the correlation between random effects. The model's estimates and standard errors are expressed in logit units. The intercept estimate (logit: -1.18000) indicates that test words were skipped in about 23% of the cases, when all variables were at their reference, mean, value (Word Length: 5.82 letters; Launch Site: -2.93 letters; Word Frequency: 3.06 log units; Word Predictability: -0.96 logit units). Colon stands for interaction.
(DOCX)

S4 Table Fixed effects of minimalist optimal GLMM (Model 2’) for the probability of word skipping. This analysis was conducted across all words in the sentences that responded to our selection criteria (see Materials and Methods). The fixed structure included the effects of word length (“LENGTH”; 4–8 letters), word frequency (“FREQ”; between 0.01 and 9.02 log units), and saccadic launch-site distance (“LAUNCH”; between -6.00 and -0.002 letters from the space in front of the test words), as well as all three- and two-way interactions, except for the interaction between word length and launch site; the random structure included a random intercept by participant, sentence pair, and word, as well as by-participant random effects of word length and saccadic launch-site distance (see S1 Table). The model's estimates and standard errors are expressed in logit units. The intercept estimate (logit: -69274) indicates that the words were skipped in about 33% of the cases, when all variables were at their reference, mean, value (Word Length: 5.60 letters; Launch Site: -2.40 letters; Word Frequency: 4.33 log units). Colon stands for interaction.
(DOCX)

S5 Table Fixed effects of minimalist optimal LMM for initial landing positions in the test words. Initial eye landing positions were expressed in letters relative to the center of the test words. The fixed structure included the effects of word length (“LENGTH”; 3–11 letters), and saccadic launch-site distance (“LAUNCH”; between -8.00 and -0.002 letters from the space in front of the test words), the two-way interactions between word frequency and word length, word predictability and word length, and word length and launch site, as well as the three-way interaction between word frequency, word length and launch site; the random structure included a random intercept by participant and sentence pair, as well as by-participant random effects of word length, word predictability and saccadic launch-site distance (see S1 Table). The intercept estimate gives the initial landing position when all variables were at their reference, mean, value (Word Length: 6.20 letters; Launch Site: -4.39 letters; Word Frequency: 2.91 log units; Word Predictability: -0.97 logit units). Colon stands for interaction.
(DOCX)

S6 Table Fixed effects of (minimalist) optimal LMM for initial landing positions in the test words, for comparison with McConkie et al.’s [18] findings. Initial eye landing positions were expressed in letters relative to the center of the test words. In the optimal models (a,c), the fixed structure included the effects of word length (“LENGTH”; 3–11 letters (a); 4–8 letters (c)) and saccadic launch-site distance (“LAUNCH”; between -12 and -4 letters from the center of the test words), as well as the interaction, and in the minimalist optimal models (b,d), the fixed structure comprised only the effect of saccadic launch-site distance and the interaction between word length (3–11 letters (b); 4–8 letters (d)) and launch site. The random structure included a random intercept by participant and by sentence pair, as well as by-participants random effects of word length and launch site. The intercept estimate gives the initial landing position when all variables were at their reference, mean, value (Word Length: 6.01 letters (a); 5.85 letters (b); Launch Site: -8.45 letters (a); -8.42 letters (b)). Colon stands for interaction.
(DOCX)

S7 Table Fixed effects of minimalist optimal LMM for within-word initial landing positions. This analysis was conducted across all words in the sentences that responded to our selection criteria (see Materials and Methods). Within-word initial landing positions were expressed in letters relative to the center of words. The fixed structure included an effect of word length (“LENGTH”; 3–11 letters), word frequency (“FREQ”; between -2.66 and 9.59 log units), and saccadic launch-site distance (“LAUNCH”; between -9.99 and -0.001 letters from the space in front of the words), as well as all interactions, except for the interaction between word frequency and launch site; the random structure included a random intercept by participant, sentence pair, and word, as well as by-participant random effects of word frequency, word length and launch-site distance (see S1 Table). The intercept estimate gives the initial landing position when all variables were at their reference, mean, value (Word Length: 5.94 letters; Launch Site: -4.86 letters; Word Frequency: 4.11 log units). Colon stands for interaction.
(DOCX)

S8 Table Fixed effects of optimal LMM for within-word initial landing positions, for comparison with McConkie et al.’s [18] findings. These analyses were conducted using all words in the sentences that responded to our selection criteria (see Materials and Methods). Initial eye landing positions were expressed in letters relative to the center of words. The fixed structure included the effects of word length (“LENGTH”; 3–11 letters (a); 4–8 letters (b)) and saccadic launch-site distance (“LAUNCH”; between -12 and -4 letters from the words’ center), as well as the interaction; the random structure included a random intercept by participant, sentence pair, and word, as well as by-participant random effects of word length and launch-site distance. The intercept estimate gives the initial landing position when all variables were at their reference, mean, value (Word Length: 5.79 letters (a); 5.88 letters (b); Launch Site: -8.29 letters (a); -8.22 letters (b)). Colon stands for interaction. Note that corresponding minimalist optimal models were exactly identical.
(DOCX)

S9 Table Fixed effects of optimal LMM for overall landing positions, for comparison with McConkie et al.’s [18] findings. Were considered for analysis all saccades’ landing positions; these were expressed relative to the center of Word N+1. The fixed structure included the effects of word length (“LENGTH”; 3–11 letters) and saccadic launch-site distance (“LAUNCH”; between -12 and -2.50 letters from the center of Word N+1), as well as the interaction. The random structure included a random intercept by participant, sentence pair, and word, as well as by-participant random effects of word length and saccadic launch-site distance. The intercept estimate gives the landing position when all variables were at their reference, mean, value (Word Length: 5.01 letters; Launch Site: -6.65 letters). Colon stands for interaction. Note that the corresponding minimalist optimal model was exactly identical.
(DOCX)
