# Supplementary material for: Linguistic processes do not beat visuo-motor constraints, but they modulate where the eyes move regardless of word boundaries: Evidence against top-down word-based eye-movement control during reading
Source: PLoS One. 2019 Jul 22;14(7):e0219666. doi: 10.1371/journal.pone.0219666 (PMC6645505; doi:10.1371/journal.pone.0219666)
Supplement: S1 Table — When optimal and minimalist optimal (G)LMMs for a given analysis were identical, only the optimal model’s formula was reported. SKIP was a binary variable, indicating whether, or not, the (target) word was skipped. LP corresponded to within-word landing positions, and OVLP corresponded to saccades’ overall landing positions. SENTENCE corresponded to Sentence Pair, and WORD corresponded to Word Number. See the corresponding tables’ legends for definition of the predictor variables. (DOCX) [file pone.0219666.s001.docx]

| **TABLES** | **FORMULAS** |
| --- | --- |
| **2** | $GLMM1=glmer (SKIP \sim FREQ*LENGTH+\left( 1+LENGTH \vert SUBJECT \right)+\left( 1 \vert SENTENCE \right))$ |
| **S2** | $GLMM1=glmer (SKIP \sim FREQ:LENGTH+LENGTH+\left( 1+LENGTH \vert SUBJECT \right)+\left( 1 \right\vert SENTENCE))$ |
| **3** | $GLMM2=glmer (SKIP \sim PRED*LENGTH*LAUNCH+FREQ*LENGTH*LAUNCH+(1+LENGTH+LAUNCH \vert\vert SUBJECT) +(1 \vert SENTENCE))$ |
| **S3** | $GLMM2=glmer (SKIP \sim PRED:LENGTH:LAUNCH+FREQ:LENGTH:LAUNCH+LENGTH+LAUNCH+\left( 1+LENGTH+LAUNCH \vert\vert SUBJECT \right)+(1 \vert SENTENCE)$) |
| **4** | $GLMM1^{'}=glmer (SKIP \sim FREQ*LENGTH+(1+FREQ+LENGTH \vert\vert SUBJECT) +\left( 1 \vert SENTENCE \right)+(1 \vert WORD))$ |
| **5** | $GLMM2^{'}=glmer (SKIP \sim FREQ*LENGTH*LAUNCH+\left( 1+LENGTH+LAUNCH \vert SUBJECT \right)+\left( 1 \vert SENTENCE \right)+\left( 1 \vert WORD \right))$ |
| **S4** | $GLMM2^{'}=glmer (SKIP \sim FREQ:LENGTH:LAUNCH+FREQ:LENGTH+FREQ:LAUNCH+FREQ+LENGTH+LAUNCH+\left( 1+LENGTH+LAUNCH \vert SUBJECT \right)+\left( 1 \vert SENTENCE \right)+\left( 1 \vert WORD \right))$ |
| **6** | $LMM=lmer (LP \sim FREQ*LENGTH*LAUNCH+PRED:LENGTH+PRED+\left( 1+LENGTH+LAUNCH+PRED \vert SUBJECT \right)+\left( 1 \vert SENTENCE \right))$ |
| **S5** | $LMM=lmer (LP \sim FREQ:LENGTH:LAUNCH+FREQ:LENGTH+PRED:LENGTH+LENGTH:LAUNCH+LENGTH+LAUNCH+\left( 1+LENGTH+LAUNCH+PRED \vert SUBJECT \right)+\left( 1 \vert SENTENCE \right))$ |
| **7** | $LMM=lmer (LP \sim FREQ*LENGTH*LAUNCH+\left( 1+LENGTH+LAUNCH+FREQ \vert SUBJECT \right)+\left( 1 \vert SENTENCE \right)+\left( 1 \vert WORD \right))$ |
| **S7** | $LMM=lmer (LP \sim FREQ:LENGTH:LAUNCH+FREQ:LENGTH+LENGTH:LAUNCH+FREQ+ LENGTH+LAUNCH+\left( 1+LENGTH+LAUNCH+FREQ \vert SUBJECT \right)+\left( 1 \vert SENTENCE \right)+\left( 1 \vert WORD \right))$ |
| **8** | $LMM=lmer (OVLP \sim FREQ*LENGTH+LAUNCH+\left( 1+LENGTH+LAUNCH+FREQ \vert SUBJECT \right)+\left( 1 \vert SENTENCE \right)+\left( 1 \vert WORD \right))$ |
