# Supplementary material for: Linguistic processes do not beat visuo-motor constraints, but they modulate where the eyes move regardless of word boundaries: Evidence against top-down word-based eye-movement control during reading
Source: PLoS One. 2019 Jul 22;14(7):e0219666. doi: 10.1371/journal.pone.0219666 (PMC6645505; doi:10.1371/journal.pone.0219666)
Supplement: S2 Table — The optimal fixed structure included the effect of word length (“LENGTH”; 3–11 letters) and the interaction between word length and word frequency (“FREQ”; 0.20–5.93 log units; the optimal random structure included a random intercept by participant and sentence pair, as well as a random effect of word length by participant (see S1 Table). The model's estimates and standard errors are expressed in logit units. The intercept estimate (logit: -1.59253) indicates that the probability of word skipping was of about 0.17 when all variables were at their reference, mean, value (Word Length: 5.96 letters; Word Frequency: 3.03 log units). Colon stands for interaction. (DOCX) [file pone.0219666.s002.docx]

|  | **Estimate** | **Std. Error** | **z value** | **Pr(>\|z\|)** |
| --- | --- | --- | --- | --- |
| **(Intercept)** | -1.59253 | 0.08930 | -17.83304 | < 0.00001 |
| **LENGTH** | -0.35671 | 0.02694 | -13.24125 | < 0.00001 |
| **FREQ:LENGTH** | -0.03052 | 0.01297 | -2.35346 | 0.01860 |
