# Supplementary material for: Linguistic processes do not beat visuo-motor constraints, but they modulate where the eyes move regardless of word boundaries: Evidence against top-down word-based eye-movement control during reading
Source: PLoS One. 2019 Jul 22;14(7):e0219666. doi: 10.1371/journal.pone.0219666 (PMC6645505; doi:10.1371/journal.pone.0219666)
Supplement: S3 Table — The optimal fixed structure included the effects of word length (“LENGTH”; 4–8 letters), and saccadic launch-site distance (“LAUNCH”; between -6.00 and -0.002 letters from the space in front of the test words), and the three-way interactions between word frequency, word length and launch-site distance and between word predictability, word length and launch-site distance; the random structure included a random intercept by participant and by sentence pair, as well as by-participant random effects of word length and launch-site distance, but without the correlation between random effects. The model's estimates and standard errors are expressed in logit units. The intercept estimate (logit: -1.18000) indicates that test words were skipped in about 23% of the cases, when all variables were at their reference, mean, value (Word Length: 5.82 letters; Launch Site: -2.93 letters; Word Frequency: 3.06 log units; Word Predictability: -0.96 logit units). Colon stands for interaction. (DOCX) [file pone.0219666.s003.docx]

|  | **Estimate** | **Std. Error** | **z value** | **Pr(>\|z\|)** |
| --- | --- | --- | --- | --- |
| **(Intercept)** | -1.18000 | 0.14775 | -7.98624 | < 0.00001 |
| **LENGTH** | -0.45068 | 0.05592 | -8.05975 | < 0.00001 |
| **LAUNCH** | 0.61378 | 0.04084 | 15.02743 | < 0.00001 |
| **PRED:LENGTH:LAUNCH** | 0.01444 | 0.01098 | 1.31532 | 0.18840 |
| **FREQ:LENGTH:LAUNCH** | 0.02497 | 0.01309 | 1.90765 | 0.05644 |
