# Supplementary material for: Linguistic processes do not beat visuo-motor constraints, but they modulate where the eyes move regardless of word boundaries: Evidence against top-down word-based eye-movement control during reading
Source: PLoS One. 2019 Jul 22;14(7):e0219666. doi: 10.1371/journal.pone.0219666 (PMC6645505; doi:10.1371/journal.pone.0219666)
Supplement: S4 Table — This analysis was conducted across all words in the sentences that responded to our selection criteria (see Materials and Methods). The fixed structure included the effects of word length (“LENGTH”; 4–8 letters), word frequency (“FREQ”; between 0.01 and 9.02 log units), and saccadic launch-site distance (“LAUNCH”; between -6.00 and -0.002 letters from the space in front of the test words), as well as all three- and two-way interactions, except for the interaction between word length and launch site; the random structure included a random intercept by participant, sentence pair, and word, as well as by-participant random effects of word length and saccadic launch-site distance (see S1 Table). The model's estimates and standard errors are expressed in logit units. The intercept estimate (logit: -69274) indicates that the words were skipped in about 33% of the cases, when all variables were at their reference, mean, value (Word Length: 5.60 letters; Launch Site: -2.40 letters; Word Frequency: 4.33 log units). Colon stands for interaction. (DOCX) [file pone.0219666.s004.docx]

|  | **Estimate** | **Std. Error** | **z value** | **Pr(>\|z\|)** |
| --- | --- | --- | --- | --- |
| **(Intercept)** | -0.69274 | 0.13471 | -5.14250 | < 0.00001 |
| **FREQ** | 0.07125 | 0.01617 | 4.40554 | 0.00001 |
| **LENGTH** | -0.68575 | 0.04195 | -16.34779 | < 0.00001 |
| **LAUNCH** | 0.61224 | 0.04291 | 14.26961 | < 0.00001 |
| **FREQ:LENGTH** | -0.06144 | 0.01035 | -5.93765 | < 0.00001 |
| **FREQ:LAUNCH** | -0.01368 | 0.00690 | -1.98127 | 0.04756 |
| **FREQ:LENGTH:LAUNCH** | 0.01127 | 0.00540 | 2.08816 | 0.03678 |
