# Supplementary material for: Linguistic processes do not beat visuo-motor constraints, but they modulate where the eyes move regardless of word boundaries: Evidence against top-down word-based eye-movement control during reading
Source: PLoS One. 2019 Jul 22;14(7):e0219666. doi: 10.1371/journal.pone.0219666 (PMC6645505; doi:10.1371/journal.pone.0219666)
Supplement: S5 Table — Initial eye landing positions were expressed in letters relative to the center of the test words. The fixed structure included the effects of word length (“LENGTH”; 3–11 letters), and saccadic launch-site distance (“LAUNCH”; between -8.00 and -0.002 letters from the space in front of the test words), the two-way interactions between word frequency and word length, word predictability and word length, and word length and launch site, as well as the three-way interaction between word frequency, word length and launch site; the random structure included a random intercept by participant and sentence pair, as well as by-participant random effects of word length, word predictability and saccadic launch-site distance (see S1 Table). The intercept estimate gives the initial landing position when all variables were at their reference, mean, value (Word Length: 6.20 letters; Launch Site: -4.39 letters; Word Frequency: 2.91 log units; Word Predictability: -0.97 logit units). Colon stands for interaction. (DOCX) [file pone.0219666.s005.docx]

|  | **Estimate** | **Std. Error** | **t value** |
| --- | --- | --- | --- |
| **(Intercept)** | -0.55479 | 0.11489 | -4.82905 |
| **LENGTH** | -0.20748 | 0.02174 | -9.54195 |
| **LAUNCH** | 0.43018 | 0.02479 | 17.35466 |
| **FREQ:LENGTH** | 0.02707 | 0.00921 | 2.93995 |
| **PRED:LENGTH** | 0.01457 | 0.00635 | 2.29387 |
| **LENGTH:LAUNCH** | 0.04451 | 0.00492 | 9.04310 |
| **FREQ:LENGTH:LAUNCH** | 0.00519 | 0.00298 | 1.74291 |
