# Supplementary material for: Linguistic processes do not beat visuo-motor constraints, but they modulate where the eyes move regardless of word boundaries: Evidence against top-down word-based eye-movement control during reading
Source: PLoS One. 2019 Jul 22;14(7):e0219666. doi: 10.1371/journal.pone.0219666 (PMC6645505; doi:10.1371/journal.pone.0219666)
Supplement: S6 Table — Initial eye landing positions were expressed in letters relative to the center of the test words. In the optimal models (a,c), the fixed structure included the effects of word length (“LENGTH”; 3–11 letters (a); 4–8 letters (c)) and saccadic launch-site distance (“LAUNCH”; between -12 and -4 letters from the center of the test words), as well as the interaction, and in the minimalist optimal models (b,d), the fixed structure comprised only the effect of saccadic launch-site distance and the interaction between word length (3–11 letters (b); 4–8 letters (d)) and launch site. The random structure included a random intercept by participant and by sentence pair, as well as by-participants random effects of word length and launch site. The intercept estimate gives the initial landing position when all variables were at their reference, mean, value (Word Length: 6.01 letters (a); 5.85 letters (b); Launch Site: -8.45 letters (a); -8.42 letters (b)). Colon stands for interaction. (DOCX) [file pone.0219666.s006.docx]

| **(a)** | **Estimate** | **Std. Error** | **t value** |
| --- | --- | --- | --- |
| **(Intercept)** | -0.49515 | 0.11149 | -4.44125 |
| **LENGTH** | -0.00153 | 0.02123 | -0.07228 |
| **LAUNCH** | 0.41751 | 0.02483 | 16.81355 |
| **LENGTH:LAUNCH** | 0.05867 | 0.00538 | 10.90400 |

| **(b)** | **Estimate** | **Std. Error** | **t value** |
| --- | --- | --- | --- |
| **(Intercept)** | -0.49023 | 0.08927 | -5.49168 |
| **LAUNCH** | 0.41767 | 0.02471 | 16.90053 |
| **LENGTH:LAUNCH** | 0.05866 | 0.00537 | 10.92620 |

| **(c)** | **Estimate** | **Std. Error** | **t value** |
| --- | --- | --- | --- |
| **(Intercept)** | -0.47362 | 0.11200 | -4.22856 |
| **LENGTH** | 0.01233 | 0.02582 | 0.47741 |
| **LAUNCH** | 0.41300 | 0.02495 | 16.55580 |
| **LENGTH:LAUNCH** | 0.06472 | 0.00710 | 9.11411 |

| **(d)** | **Estimate** | **Std. Error** | **t value** |
| --- | --- | --- | --- |
| **(Intercept)** | -0.50173 | 0.09462 | -5.30277 |
| **LAUNCH** | 0.41217 | 0.02489 | 16.56121 |
| **LENGTH:LAUNCH** | 0.06448 | 0.00709 | 9.09073 |
