# Supplementary material for: Linguistic processes do not beat visuo-motor constraints, but they modulate where the eyes move regardless of word boundaries: Evidence against top-down word-based eye-movement control during reading
Source: PLoS One. 2019 Jul 22;14(7):e0219666. doi: 10.1371/journal.pone.0219666 (PMC6645505; doi:10.1371/journal.pone.0219666)
Supplement: S7 Table — This analysis was conducted across all words in the sentences that responded to our selection criteria (see Materials and Methods). Within-word initial landing positions were expressed in letters relative to the center of words. The fixed structure included an effect of word length (“LENGTH”; 3–11 letters), word frequency (“FREQ”; between -2.66 and 9.59 log units), and saccadic launch-site distance (“LAUNCH”; between -9.99 and -0.001 letters from the space in front of the words), as well as all interactions, except for the interaction between word frequency and launch site; the random structure included a random intercept by participant, sentence pair, and word, as well as by-participant random effects of word frequency, word length and launch-site distance (see S1 Table). The intercept estimate gives the initial landing position when all variables were at their reference, mean, value (Word Length: 5.94 letters; Launch Site: -4.86 letters; Word Frequency: 4.11 log units). Colon stands for interaction. (DOCX) [file pone.0219666.s007.docx]

|  | **Estimate** | **Std. Error** | **t value** |
| --- | --- | --- | --- |
| **(Intercept)** | -0.62166 | 0.08112 | -7.66339 |
| **FREQ** | 0.01977 | 0.01009 | 1.95839 |
| **LENGTH** | -0.22463 | 0.01653 | -13.58700 |
| **LAUNCH** | 0.36060 | 0.01666 | 21.64899 |
| **FREQ:LENGTH** | 0.01680 | 0.00246 | 6.83735 |
| **LENGTH:LAUNCH** | 0.04680 | 0.00187 | 25.04487 |
| **FREQ:LENGTH:LAUNCH** | 0.00443 | 0.00069 | 6.41646 |
