# Supplementary material for: Linguistic processes do not beat visuo-motor constraints, but they modulate where the eyes move regardless of word boundaries: Evidence against top-down word-based eye-movement control during reading
Source: PLoS One. 2019 Jul 22;14(7):e0219666. doi: 10.1371/journal.pone.0219666 (PMC6645505; doi:10.1371/journal.pone.0219666)
Supplement: S8 Table — These analyses were conducted using all words in the sentences that responded to our selection criteria (see Materials and Methods). Initial eye landing positions were expressed in letters relative to the center of words. The fixed structure included the effects of word length (“LENGTH”; 3–11 letters (a); 4–8 letters (b)) and saccadic launch-site distance (“LAUNCH”; between -12 and -4 letters from the words’ center), as well as the interaction; the random structure included a random intercept by participant, sentence pair, and word, as well as by-participant random effects of word length and launch-site distance. The intercept estimate gives the initial landing position when all variables were at their reference, mean, value (Word Length: 5.79 letters (a); 5.88 letters (b); Launch Site: -8.29 letters (a); -8.22 letters (b)). Colon stands for interaction. Note that corresponding minimalist optimal models were exactly identical. (DOCX) [file pone.0219666.s008.docx]

| **(a)** | **Estimate** | **Std. Error** | **t value** |
| --- | --- | --- | --- |
| **(Intercept)** | -0.40048 | 0.07990 | -5.01226 |
| **LENGTH** | -0.03458 | 0.01972 | -1.75357 |
| **LAUNCH** | 0.35547 | 0.01938 | 18.34606 |
| **LENGTH:LAUNCH** | 0.06139 | 0.00260 | 23.60626 |

| **(b)** | **Estimate** | **Std. Error** | **t value** |
| --- | --- | --- | --- |
| **(Intercept)** | -0.39095 | 0.08632 | -4.52927 |
| **LENGTH** | -0.04200 | 0.02208 | -1.90260 |
| **LAUNCH** | 0.37422 | 0.02121 | 17.64423 |
| **LENGTH:LAUNCH** | 0.06772 | 0.00430 | 15.74322 |
