# Supplementary material for: Linguistic processes do not beat visuo-motor constraints, but they modulate where the eyes move regardless of word boundaries: Evidence against top-down word-based eye-movement control during reading
Source: PLoS One. 2019 Jul 22;14(7):e0219666. doi: 10.1371/journal.pone.0219666 (PMC6645505; doi:10.1371/journal.pone.0219666)
Supplement: S9 Table — Were considered for analysis all saccades’ landing positions; these were expressed relative to the center of Word N+1. The fixed structure included the effects of word length (“LENGTH”; 3–11 letters) and saccadic launch-site distance (“LAUNCH”; between -12 and -2.50 letters from the center of Word N+1), as well as the interaction. The random structure included a random intercept by participant, sentence pair, and word, as well as by-participant random effects of word length and saccadic launch-site distance. The intercept estimate gives the landing position when all variables were at their reference, mean, value (Word Length: 5.01 letters; Launch Site: -6.65 letters). Colon stands for interaction. Note that the corresponding minimalist optimal model was exactly identical. (DOCX) [file pone.0219666.s009.docx]

|  | **Estimate** | **Std. Error** | **t value** |
| --- | --- | --- | --- |
| **(Intercept)** | 1.97654 | 0.21888 | 9.03046 |
| **LENGTH** | -0.05647 | 0.02456 | -2.29944 |
| **LAUNCH** | 0.93986 | 0.02523 | 37.24650 |
| **LENGTH:LAUNCH** | 0.01293 | 0.00455 | 2.84331 |
